# Plasma lipid species at type 1 diabetes onset predict residual beta-cell function after 6 months

**DOI:** 10.1007/s11306-018-1456-3

**Published:** 2018-12-04

**Authors:** Anne Julie Overgaard, Jacquelyn M. Weir, Kaushala Jayawardana, Henrik Bindesbøl Mortensen, Flemming Pociot, Peter J. Meikle

**Affiliations:** 1 Steno Diabetes Center Copenhagen, Niels Steensensvej 2, 2820 Gentofte, Denmark; 2 Baker IDI Heart and Diabetes Research Institute, 75 Commercial Road, Melbourne, Australia; 3 Faculty of Health and Medical Sciences, University of Copenhagen, Copenhagen, Denmark; 4 Department of Biochemistry and Molecular Biology, University of Melbourne, Melbourne, Australia

**Keywords:** Prediction and prevention of type 1 diabetes, Metabonomics, Diabetes in childhood

## Abstract

**Introduction:**

The identification of metabolomic dysregulation appears promising for the prediction of type 1 diabetes and may also reveal metabolic pathways leading to beta-cell destruction. Recent studies indicate that regulation of multiple phospholipids precede the presence of autoantigens in the development of type 1 diabetes.

**Objectives:**

We hypothesize that lipid biomarkers in plasma from children with recent onset type 1 diabetes will reflect their remaining beta-cell function and predict future changes in beta-cell function.

**Methods:**

We performed targeted lipidomic profiling by electrospray ionization tandem mass spectrometry to acquire comparative measures of 354 lipid species covering 25 lipid classes and subclasses in plasma samples from 123 patients < 17 years of age followed prospectively at 1, 3, 6 and 12 months after diagnosis. Lipidomic profiles were analysed using liner regression to investigate the relationship between plasma lipids and meal stimulated C-peptide levels at each time point. P-values were corrected for multiple comparisons by the method of Benjamini and Hochberg.

**Results:**

Linear regression analysis showed that the relative levels of cholesteryl ester, diacylglycerol and triacylglycerol at 1 month were associated to the change in c-peptide levels from 1 to 6 months (corrected p-values of 4.06E−03, 1.72E−02 and 1.72E02, respectively). Medium chain saturated and monounsaturated fatty acids were the major constituents of the di- and triacylglycerol species suggesting a link with increased lipogenesis.

**Conclusion:**

These observations support the hypothesis of lipid disturbances as explanatory factors for residual beta-cell function in children with new onset type 1 diabetes.

**Electronic supplementary material:**

The online version of this article (10.1007/s11306-018-1456-3) contains supplementary material, which is available to authorized users.

## Introduction

The incidence of type 1 diabetes in childhood is increasing worldwide (Patterson et al. 2009; Soltesz et al. 2007). Across Europe the incidence rate of type 1 diabetes in childhood is increasing with approximately 3–4% per year (Patterson et al. 2012; Svensson et al. 2009). The exact mechanisms triggering and regulating progression towards beta-cell failure in type 1 diabetes are poorly understood but it is generally acknowledged that both genetic and environmental components are involved (Rewers and Ludvigsson 2016). Within a short time after disease onset approximately half of the patients will experience a remission phase or honeymoon period characterized by endogenous insulin production and an improved glycemic control (von Herrath et al. 2007). This has, been labelled by some (Muhammad et al. 1999), a ‘window of opportunity’ for treatment towards preventing the further immune destruction of the beta-cells or even restoration of the beta-cells.

Lipidomics systematically reflects the detectable lipids of cells, tissues and biofluids to capture a lipid snapshot of a given physiological and environmental milieu. The lipidomic phenotype is sensitive to subtle modifications in e.g. lifestyle and nutrition (Zheng and Qi 2014), and accordingly changes in the lipidome will reflect both genetic and environmental factors, influencing susceptibility to developing chronic disease such as type 1 diabetes. Advanced technologies allow high-throughput profiling of the lipidome from a low volume blood specimen, and focus has been towards establishing lipidomic profiles that can predict disease status and progression (Meikle et al. 2011; Weir et al. 2013). Recent research of the lipidome in relation to type 1 diabetes indicates that modulation of multiple serum phospholipids precedes the presence of autoantigens in the development of type 1 diabetes (Pflueger et al. 2011; Oresic et al. 2008). Children progressing to type 1 diabetes before 4 years of age had lower levels of phospholipids in their cord blood (La Torre et al. 2013). These observations suggest the potential of lipidomic biomarker panels in monitoring beta-cell function. Thus, we hypothesize that new biomarkers in the form of lipidomic panels in plasma from children with recent onset type 1 diabetes can predict decline or regeneration of the endogenous residual beta-cell function and may serve as markers for decline in beta-cell function before type 1 diabetes manifests clinically.

In this study we profiled the plasma lipidome of a cohort of children diagnosed with type 1 diabetes and followed in the Danish Remission Phase Study. The objective of the study was to identify lipidomic profiles associated with the progression of the disease as assessed by changes in meal stimulated C-peptide levels within the first year after type 1 diabetes onset.

## Materials and methods

### Study subjects

The Danish Remission Phase Study is a prospective long-term observational study conducted in four pediatric departments in Denmark. Samples from a total of 123 children and adolescents aged less than 17 years with newly diagnosed type 1 diabetes enrolled from April 2004 to August 2006 and were followed for 12 months from onset of type 1 diabetes, defined as the first insulin injection, were analyzed. The detailed study design has previously been described by Andersen et al. (2012). The study was performed according to the criteria of the Helsinki II Declaration and was approved by the Danish National Committee on Biomedical Research Ethics (Journal Number: H-KA-04010-m). Older patients and all parents or guardians gave written informed consent. Data were transmitted anonymously by the centers; patients were identified by center number and patient code. Plasma samples from 1, 3, 6 and 12 months were analysed.

### Residual beta-cell function (stimulated C-peptide)

Stimulated serum C-peptide was used as a marker of endogenous C-peptide release 1, 3, 6 and 12 after diagnosis (± 1 week) (Table 1). Stimulated C-peptide measurements under standardized conditions can be used as a clinically validated measurement of beta-cell function, as C-peptide is co-secreted in a one-to-one molar ratio to insulin (Palmer et al. 2004). There were 123 patients who contributed with at least one measurement during the first year. Blood was drawn 90 min after the ingestion of a liquid meal [Boost drink, formerly known as Sustacal (237 mL or 8 FL OZ containing 41 g carbohydrate, 10 g protein and 4 g fat, 240 kcal): 6 mL/kg (maximal 360 mL), Novartis Medical Health, Inc., Minneapolis, MN, USA, http://www.boost.com] according to Diabetes Control and Complication trial (DCCT) standards (The Diabetes Control and Complications Trial Research Group 1998). Serum samples were labeled and frozen at − 80 °C until shipment on dry ice. Samples were thawed twice for the C-peptide assay and aliquoting. Serum C-peptide was analyzed centrally using a fluoroimmunometric assay after adding a standard as described thoroughly by Mortensen et al. (2010).

**Table 1:** Clinical characteristic at the time of blood sampling

	1 month	3 months	6 months	12 months
N (%male)	123 (52)	118 (51)	111 (53)	116 (51)
C-peptide (pmol/L)*	703.36 (395.02)	691.19 (445.29)	589.22 (417.29)	396.43 (371.43)
Height (cm)	144.38 (24.53)	145.33 (24.94)	147.84 (23.37)	149.61 (24.38)
Age (years)	10.08 (3.90)	10.11 (3.92)	10.34 (3.81)	10.17 (3.94)
Weight (kg)	39.79 (16.68)	40.71 (16.86)	42.41 (17.16)	43.79 (17.64)
Blood glucose after meal stimulation (pmol/L)*	14.56 (3.96)	15.34 (4.50)	17.86 (5.14)	19.93 (5.04)
BMI (kg/m^2^)	18.17 (2.77)	18.25 (2.78)	18.44 (3.07)	18.60 (2.93)
Hba1c (%)*	9.26 (1.20)	6.96 (0.88)	7.20 (1.21)	7.72 (1.36)

Characteristics at the time of blood samplings. Data is presented as mean (SD) where indicated

*BMI* body mass index, *HbA1c* haemoglobin A1c

*p < 0.05 in comparison between groups evaluated using ANOVA or Kruskal–Wallis

### Extraction of lipids

Lipid species were extracted from plasma samples as described previously (Alshehry et al. 2015). Briefly, plasma (10 µL) was aliquoted into a 1.5 mL eppendorf tube using a positive displacement pipette and 100 µL of 1-butanol/methanol (1:1, v/v), 5 mM ammonium formate containing internal standards (Supplementary Table 1) was added, also using a positive displacement pipette. The mixture was vortexed for 10 s, sonicated for 60 min in a sonic water bath (18–24 °C) and then centrifuged (16,000×*g*, 10 min, 20 °C). The supernatant was transferred into a 0.2 mL glass insert with Teflon insert caps for lipidomic analysis (Alshehry et al. 2015; Begum et al. 2017).

### Lipidomics

In this study lipidomic analysis was performed by liquid chromatography electrospray ionisation tandem mass spectrometry on an Agilent 1290 liquid chromatography system combined with an Agilent 6490 triple quadrupole mass spectrometer as described previously (Mundra et al. 2018). We used scheduled multiple-reaction monitoring [MRM] in positive ion mode, building upon early methods (Meikle et al. 2011; Weir et al. 2013; Wong et al. 2013). The mass spectrometer was operated in dynamic/scheduled multiple reaction monitoring (dMRM) mode. There were 353 unique lipid species measured together with 13 stable isotope or non-physiological lipid standards (Supplementary Table 1). Mass spectrometer voltages used for the acquisition of data were; fragmentor voltage, 380 V and cell accelerator voltage, 5 V. The collision energy voltage was set individually for each lipid class as reported previously (Mundra et al. 2018). There were several sets of isobaric lipids which shared the same nominal parent ion mass and also give rise to the same product ions. Specifically, for isobaric species of phosphatidylcholine, alkylphosphatidylcholine and alkenylphosphatidylcholine the parent and product ions (m/z 184) were the same. As a result a single MRM transition was used to measure the corresponding species within each subclass, using an increased MRM window time (21 combinations). A total of 335 MRMs were used. Dwell time per MRM ranged between 7 and 100 ms depending on where within the gradient the measurement were made and how many concurrent MRMs were being measured.

The total run time per sample was 11 min. The total study analysis period was 120 h. Identical plasma quality control samples, obtained by combining plasma from six healthy adults (24–54 years) prepared immediately after collection and stored at − 80° until required for analysis were distributed throughout the extraction series of the patient plasma for every 15 patient samples, to monitor assay performance. The analysis was performed in one single analytical run with no breaks. Peak integration of both standards and plasma samples were performed using the MassHunter software, Agilent Technologies, with manual inspection. Concentrations of the individual lipid species were estimated using a single point quantitation based on the area under the chromatographic peak compared to the area under the peak for the relative internal standard (known amount). The total of each lipid class was calculated from the sum of the individual lipid species within that class. Identification of individual lipid species was based on the MRM experiment and retention time (MSI level 2) and was matched to previous fragmentation analysis in positive and negative ionization mode as described in detail by Weir et al. (2013).

A total of 353 lipid species were identified and quantified from the lipid classes: dihydroceramide (dhCer), ceramide (Cer), monohexosylceramide (MHC), dihexosylceramide (DHC), trihexosylceramide (THC), GM3 ganglioside (GM3), sphingomyelin (SM), phosphatidylcholine (PC), oxidized phosphatidylcholine (OxPC), alkylphosphatidylcholine (PC(O)), alkenylphosphatidylcholine (plasmalogen, PC(P)), lysophosphatidylcholine (LPC), lysoalkylphosphatidylcholine (lysoplatelet activating factor, LPC(O)), phosphatidylethanolamine (PE), alkylphosphatidylethanolamine (PE(O)), phosphatidylethanolamine plasmalogen (PE(P)), lysophosphatidylethanolamine (LPE), phosphatidylinositol (PI), lysophosphatidylinositol (LPI), phosphatidylserine (PS), phosphatidylglycerol (PG), free cholesterol (COH), cholesteryl ester (CE), free cholesterol (COH), diacylglycerol (DG) and triacylglycerol (TG). The abbreviations listed here refer to the lipid classes and subclasses, the number of carbons and double bonds will be listed when referring to individual lipid species, such as LPC(22:6) which defines a lysophosphatidylcholine containing a fatty acid comprising 22 carbon atoms and six double bonds. Measurements of lipids composed of two fatty acids are determined as the sum of the carbons and the double bonds across both fatty acids, e.g. PC(36:4) based on previous methods (Weir et al. 2013; Meikle et al. 2013). The blood samples used in this study were collected in 2004 and have been stored at − 80 °C since, and only thawed once before the analysis, which should not have any impact on stability of most lipid species (Yao and Vance 1988; Hyötyläinen and Orešič 2015).

### Normalization of lipidomics data

Total content of phosphatidylcholine within each sample, the most abundant phospholipid species in blood, was used as an internal reference and lipids were normalized to this (concentration of individual lipid species/concentration of phosphatidylcholine. The statistical analysis of phosphatidylcholine class was performed on raw data). Before statistical analysis, lipidomic data was log 10 (hereafter log) transformed and normalized to the inter-quartile range.

### Statistical analysis

Differences between time points in clinical and anthropometric data were evaluated using ANOVA or Kruskal–Wallis in SPSS. Linear regression models were completed using MatLab or R and all models included the covariates sex, age, HbA1c and 1 month C-peptide levels, except in the cases where models evaluated change in C-peptide over time, where C-peptide levels at 1 month were left out. Correction for multiple testing was done using the Benjamini–Hochberg method (Benjamini 1995).

## Results

### Demographic and clinical characteristics

123 patients were enrolled in the study whereof 52% were male. Clinical information including anthropometric data is summarized in Table 1.

Throughout the first 12 months several of the patients had short periods of remission, defined as > 300 pmol/L meal stimulated C-peptide. The weight, height and the BMI accordingly, increased significantly throughout the study period. HbA1c levels were significantly higher at 1 month. Blood glucose levels after meal stimulation were significantly higher after 12 months duration of diabetes. C-peptide levels at 1 month were not associated with bicarbonate levels at diagnosis (as a measure of ketoacidosis) when adjusted for age and sex (data not shown).

### Lipidomic assay performance

The median percentage coefficient of variation (%CV) for the individual lipid species within the quality control samples of the analysis was 12.4% with 90% of lipids having CVs < 20%. Supplementary figure 1 depicts a Principal component analysis (PCA) plot of the QC samples.

### Lipid classes associated with change in C-peptide

Table 2 shows change in C-peptide levels from 1 month to 3, 6 and 12 months associated with an inter-quartile change from the 25 to 75 percentile of the respective lipid classes at the 1 month.

**Table 2:** Lipid classes associated with change in C-peptide adjusted for age, sex and HbA1c

Outcome	Change in C-peptide, 1 to 3 month			Change in C-peptide, 1 to 6 month			Change in C-peptide, 1 to 12 month		
Lipid class	Change in outcome (pmol/L)^a^ (95% CI)	p-value	p-value (Benjamini–Hochberg)	Change in outcome (pmol/L)^a^ (95% CI)	p-value	p-value (Benjamini–Hochberg)	Change in outcome (pmol/L)^a^ (95% CI)	p-value	p-value (Benjamini–Hochberg)
Dihydroceramide	15.9 (− 36, 68)	5.5E−01	8.0E−01	6.4 (−56, 69)	8.4E−01	9.1E−01	− 50.0 (− 135, 35)	2.5E−01	4.2E−01
Ceramide	0.6 (− 62, 64)	9.9E−01	1.0E+00	− 75.8 (− 157, 5)	7.0E−02	2.5E−01	− 112.4 (− 206, − 19)	**2.1E**−**02**	1.0E−01
Monohexosylceramide	− 62.0 (− 112, − 12)	**1.6E**−**02**	3.9E−01	− 44.4 (− 108, 19)	1.8E−01	5.2E−01	− 64.2 (− 143, 15)	1.1E−01	2.6E−01
Dihexosylceramide	− 21.6 (− 77, 34)	4.5E−01	7.7E−01	− 26.4 (− 98, 46)	4.8E−01	7.9E−01	− 74.7 (− 159, 10)	8.5E−02	2.3E−01
Trihexosylceramide	− 5.3 (− 66, 55)	8.6E−01	9.9E−01	14.0 (− 59, 87)	7.1E−01	8.0E−01	− 62.1 (− 153, 29)	1.8E−01	3.5E−01
GM3 ganglioside	− 30.0 (− 91, 31)	3.4E−01	7.7E−01	− 36.6 (− 113, 39)	3.5E−01	7.2E−01	− 100.1 (− 192, − 9)	**3.4E**−**02**	1.2E−01
Spinghomyelin	− 4.2 (− 54, 46)	8.7E−01	9.9E−01	20.2 (− 50, 90)	5.7E−01	8.0E−01	− 33.7 (− 116, 49)	4.2E−01	6.1E−01
Alkylphosphatidylcholine	13.4 (− 52, 79)	6.9E−01	9.1E−01	40.1 (− 37, 117)	3.1E−01	7.0E−01	29.0 (− 74, 132)	5.8E−01	6.9E−01
Alkenylphosphatidylcholine	31.5 (− 25, 88)	2.8E−01	7.7E−01	29.7 (− 40, 99)	4.0E−01	7.2E−01	3.0 (− 85, 90)	9.5E−01	9.6E−01
Lysophosphatidylcholine	− 24.8 (− 79, 29)	3.7E−01	7.7E−01	20.2 (− 46, 87)	5.5E−01	8.0E−01	− 1.9 (− 82, 78)	9.6E−01	9.6E−01
Lysoalkylphosphatidylcholine	− 36.5 (− 95, 22)	2.2E−01	7.7E−01	− 34.4 (− 113, 45)	4.0E−01	7.2E−01	− 75.0 (− 171, 21)	1.3E−01	2.7E−01
Phosphatidylethanolamine	− 0.5 (− 54, 53)	9.9E−01	1.0E+00	− 44.5 (− 117, 28)	2.3E−01	5.8E−01	− 24.7 (− 108, 59)	5.6E−01	6.9E−01
Alkylphosphatidylethanolamine	21.6 (− 35, 79)	4.6E−01	7.7E−01	15.1 (− 59, 89)	6.9E−01	8.0E−01	102.4 (15, 190)	**2.4E**−**02**	1.0E−-01
Alkenylphosphatidylethanolamine	25.1 (− 29, 79)	3.6E−01	7.7E−01	0.9 (− 73, 75)	9.8E−01	9.9E−01	33.5 (− 51, 118)	4.4E−01	6.1E−01
Phosphatidylglycerol	− 41.3 (− 99, 17)	1.7E−01	7.7E−01	− 93.7 (− 166, − 21)	**1.3E**−**02**	8.0E−02	− 74.1 (− 160, 11)	9.2E−02	2.3E−01
Lysophosphatidylethanolamine	− 10.0 (− 67, 47)	7.3E−01	9.1E−01	− 0.5 (− 72, 71)	9.9E−01	9.9E−01	7.0 (− 74, 88)	8.7E−01	9.6E−01
Phosphatidylinositol	− 18.2 (− 72, 35)	5.1E−01	7.9E−01	− 22.7 (− 95, 50)	5.4E−01	8.0E−01	5.4 (− 76, 87)	9.0E−01	9.6E−01
Lysophosphatidylinositol	− 22.4 (− 80, 35)	4.5E−01	7.7E−01	49.4 (− 23, 122)	1.9E−01	5.2E−01	49.9 (− 38, 138)	2.7E−01	4.2E−01
Phosphatidylserine	9.4 (− 43, 62)	7.3E−01	9.1E−01	− 13.6 (− 81, 53)	6.9E−01	8.0E−01	− 52.1 (− 133, 29)	2.1E−01	3.7E−01
Cholesterol	0.1 (− 58, 58)	1.0E+00	1.0E+00	14.0 (− 57, 85)	7.0E−01	8.0E−01	− 23.3 (− 103, 56)	5.7E−01	6.9E−01
Cholesteryl ester	− 69.4 (− 136, − 3)	**4.3E**−**02**	5.3E−01	− 167.4 (− 252, − 83)	**1.8E**−**04**	**4.6E**−**03**	− 156.7 (− 257, − 56)	**2.9E**−**03**	7.1E−02
Diacylglycerol	− 49.6 (− 118, 18)	1.6E−01	7.7E−01	− 142.5 (− 230, − 55)	**1.9E**−**03**	**1.7E**−**02**	− 129.0 (− 228, − 30)	**1.2E**−**02**	1.0E−01
Triacylglycerol	− 46.6 (− 115, 22)	1.9E−01	7.7E−01	− 144.1 (− 233, − 55)	**2.1E**−**03**	**1.7E**−**02**	− 123.0 (− 220, − 26)	**1.4E**−**02**	1.0E−01
Oxidized cholesteryl ester	−32.8 (− 90, 24)	2.6E−01	7.7E−01	− 70.6 (− 145, 4)	6.6E−02	2.5E−01	− 84.4 (− 172, 3)	6.1E−02	1.9E−01
Oxidized phosphatidylcholine	40.3 (− 19, 100)	1.9E−01	7.7E−01	91.2 (17, 165)	**1.8E**−**02**	8.9E−02	105.9 (17, 194)	**2.1E**−**02**	1.0E−01

Bold values indicate statistical significance at p < 0.05

^a^Associated with an IQR Increase in Predictor (1 month lipid) concentration

Cholesteryl ester, triacylglycerol and diacylglycerol levels at 1 month were significant predictors of a decrease in C-peptide over 6 months and after 12 months however at 12 months the associations did not maintain significance after correction for multiple testing. Oxidized phosphatidylcholine levels at 1 month were predicting an increase in C-peptide after 6 and 12 months although this was not significant after Benjamini–Hochberg correction. Furthermore, ceramide and GM3 ganglioside predicted a decrease in C-peptide after 12 months; phosphatidylglycerol predicted a decrease in C-peptide after 6 months and alkylphosphatidylethanolamine an increase in C-peptide after 12 months but again, did not maintain significance after correction. Monohexocylceramide and cholesteryl ester were predictors of a decrease in C-peptide after 3 months before correction for multiple testing.

### Lipid species associated with change in C-peptide

Nine diacylglycerol species, seven of which were carrying 16:1 and/or 18:1 fatty acids, were associated with a decrease in C-peptide after 6 months (Table 3).

**Table 3:** Lipid species associated with change in C-peptide adjusted for age, sex and HbA1c

Lipid species	Change in C-peptide, 1 to 6 month		
	Change in outcome (pmol/L)^a^ (95% CI)	p-value	p-value (Benjamini–Hochberg)
CE(16:1)	− 140.9 (− 220, − 62)	7.4E−04	2.7E−02
CE(16:2)	− 134.2 (− 221, − 47)	3.2E−03	4.7E−02
CE(18:1)	− 122.8 (− 199, − 47)	2.1E−03	4.3E−02
CE(18:2)	− 153.6 (− 228, − 79)	1.1E−04	1.3E−02
CE(18:3)	− 150.8 (− 236, − 66)	7.7E−04	2.7E−02
CE(22:5)	− 123.6 (− 199, − 48)	1.8E−03	4.3E−02
CE(22:6)	− 109.7 (− 181, − 38)	3.3E−03	4.7E−02
DG(14:0/16:0)	− 147.5 (− 234, − 61)	1.1E−03	3.2E−02
DG(14:0/16:1)	− 173.6 (− 253, − 95)	3.8E−05	7.4E−03
DG(14:0/18:1)	− 162.4 (− 257, − 68)	1.1E−03	3.2E−02
DG(14:0/18:2)	− 148.4 (− 240, − 57)	2.0E−03	4.3E−02
DG(16:0/16:1)	− 159.6 (− 239, − 80)	1.6E−04	1.4E−02
DG(16:0/18:1)	− 135.3 (− 216, − 55)	1.4E−03	3.7E−02
DG(16:1/16:1)	− 191.7 (− 279, − 104)	4.2E−05	7.4E−03
DG(16:1/18:1)	− 165.5 (− 250, − 81)	2.1E−04	1.5E−02
DG(18:1/18:1)	− 130.2 (− 213, − 47)	2.7E−03	4.6E−02
TG(14:0/16:1/18:1)	− 156.7 (− 258, − 55)	3.1E−03	4.7E−02
TG(14:0/16:1/18:2)	− 151.5 (− 243, − 60)	1.6E−03	4.0E−02
TG(14:1/16:1/18:0)	− 171.9 (− 269, − 75)	7.1E−04	2.7E−02
TG(16:0/16:1/18:1)	− 154.3 (− 252, − 57)	2.5E−03	4.6E−02
TG(16:1/16:1/16:1)	− 175.6 (− 274, − 77)	7.1E−04	2.7E−02
TG(16:1/16:1/18:1)	− 167.7 (− 261, − 74)	6.5E−04	2.7E−02
TG(16:1/18:1/18:1)	− 129.0 (− 213, − 45)	3.2E−03	4.7E−02
TG(16:1/18:1/18:2)	− 120.9 (− 198, − 44)	2.7E−03	4.6E−02

^a^Associated with an IQR increase in Predictor (1 month lipid) concentration

Eight triacylglycerols were also associated with a decrease in C-peptide after 6 months. All of the triacylglycerols contained at least one 16:1 or 18:1 fatty acid. Seven cholesteryl esters were associated with a decrease in C-peptide at 6 months. Supplementary Table 2 lists the association of all lipid species to the change in C-peptide over time.

### Lipid species associated with C-peptide levels

Linear regressions between lipid species and C-peptide, adjusted for age, sex and HbA1c were performed at each time point. At 1 month the LPC(20:2), LPC(22:5) and SM(32:0) species were significantly negatively associated to C-peptide levels after correction for multiple testing and at 1 month, cholesteryl esters were associated to lower C-peptide levels at 6 months (Table 4).

**Table 4:** Lipid classes and species at 1 month significantly associated with C-peptide levels adjusted for age, sex and HbA1c

Lipid	Outcome	Outcome (pmol/L)^a^ (95% CI)	p-value	p-value (Benjamini–Hochberg)
LPC(20:2)	C-peptide at 1 month	− 132.3 (− 196, − 69)	8.3E−05	1.5E−02
LPC(22:5)	C-peptide at 1 month	− 136.3 (− 201, − 71)	7.6E−05	1.5E−02
SM(32:0)	C-peptide at 1 month	− 149.2 (− 228, − 71)	3.0E−04	3.6E−02
Total CE	C-peptide at 6 months	− 133.1 (− 213, − 53)	1.5E−03	3.8E−02

At time points other than 1 month, regressions were also corrected for c-peptide levels at 1 month

^a^Associated with an IQR increase in Predictor (1 month lipid) concentration

### Change in lipid associated to change in C-peptide

In order to assess the relationship between changes in plasma lipids and changes in C-peptide we performed linear regression between the changes in these measures, from 1 month to each other time point. There was no significant relationship between change in lipids and change in C-peptide levels.

## Discussion

This study aimed to investigate the lipidome in relation to beta-cell function in children the first year after type 1 diabetes debut. Several lipid classes were associated with a decrease in beta-cell function after diagnosis of type 1 diabetes and the level of oxidized phosphatidylcholine could predict an increase in C-peptide over time, although significance did not remain after correction for multiple testing.

Lipids involved in energy metabolism were generally predictors of lower C-peptide levels. At 1 month, the lipid classes triacylglycerol and diacylglycerol, and several species of these, were associated with a decrease in stimulated C-peptide levels after 6 months (Table 2). Intensive insulin therapy has been shown to increase triacylglycerol and weight gain in the DCCT study (Purnell et al. 2013). In the present study higher levels of triacylglycerols were associated with a significant decrease in C-peptide over 6 months. Low triacylglycerol levels have been identified in cord-blood of children developing type 1 diabetes before 2 years of age, but were also related to shorter gestational age (La Torre et al. 2013). Short gestational age is in itself related to type 1 diabetes (Dahlquist and Kallén 1992), and it can be speculated that low levels of triacylglycerol might be an accelerator- or sign of metabolic deviations leading to type 1 diabetes at very early ages. In the present study high triacylglycerol levels at onset were associated with a decrease in stimulated C-peptide at 6 months (Table 2), indicating that triacylglycerol levels affect or reflect type 1 diabetes progression differently depending on when in the disease pathogenesis these are measured. At type 1 diabetes onset, triacylglycerols could possibly act as a metabolic accelerator for beta-cell destruction similar to lipotoxicity known from the progression of type 2 diabetes (Yang and Li 2012). Of note, all of the triacylglycerol species contained a 16:1 fatty acid and some also a 18:1 fatty acid, which are primary products of lipogenesis, further suggesting an altered metabolism leading to an increase in beta cell loss (Burns et al. 2012). At a cellular level, studies on *min6* cells and isolated mouse pancreatic islets have shown an increase in the conversion of triacylglycerols to diacylglycerols during glucose stimulation (Pearson et al. 2016). DG(16:0/18:1) is likely the most abundant product of glucose stimulated triacylglycerol hydrolysis (Pearson et al. 2016), in our study this specie was associated with a decrease in C-peptide after 6 months, indicating a potential link between glucose levels at 1 month and a significant higher decrease in c-peptide over time. The end product of hydrolysis of triacylglycerols are monoacylglycerols, unfortunately our analysis did not include these low abundance lipid species.

At 1 month, the total level of triacylglycerol was associated with both weight and age which were themselves highly correlated. To avoid adjusting for highly correlated covariates we selected age as the most appropriate covariate in this adolescent cohort.

Seven cholesteryl ester species, at 1 month, were predictors of a decrease in C-peptide after 6 months. A previous study suggests that an increase in inflammatory cytokines might facilitate the uptake of cholesteryl ester enriched lipoproteins into the tissues in otherwise normolipidemic type 1 diabetes patients, and this connection also explains the increased risk of atherogenesis in type 1 diabetes patients (Ruan et al. 2006). In this context the higher level of cholesteryl esters at 1 month that is associated with higher loss of beta-cell function, is in contradiction to previous findings where increases in inflammatory cytokines in circulation has been associated with a more severe beta-cell loss over time (Kaas et al. 2012). Nevertheless, we have not yet investigated cytokine levels in relation to stimulated C-peptide levels in this particular cohort and cannot conclude on their effect on cholesteryl esters.

Sphingolipid metabolites, such as sphingomyelin, GM3, ceramides and the precursor and metabolites of ceramide, dihydroceramide, monohexosylceramide, dihexosylceramide and trihexosylceramide, were overall associated with a lower C-peptide level after 3, 6 and 12 months (Table 2), although not reaching significance after correction for multiple testing. The sphinghomyelin SM(32:0) level at 1 month was significantly associated with C-peptide levels (Table 4). Sphingolipids modulate several beta-cell signaling pathways involved in the progression of diabetes such as apoptosis, cytokine secretion, ER to golgi trafficking, islet autoimmunity and insulin gene expression and furthermore, sphingolipid metabolism on internal membranes is also implicated in the regulation of beta-cell apoptosis. Recent advances in technology has facilitated investigating the role of ceramides in beta-cell dysfunction, and the current debate on the role of ceramides in type 1 diabetes focuses on whether ceramides can mimic the effects of IL-1β in promoting beta-cell death and in repressing insulin production (Boslem et al. 2012). The mentioned functions of sphingolipids are executed primarily within the cells, whereas our data is from lipids in circulation. Whether circulating lipid levels reflect beta cell lipid metabolism remains to be elucidated.

Two different lysophosphatidylcholine species were associated with a lower C-peptide level at 1 month (Table 4). Lower levels of lysophosphatidylcholine in cord blood in a population of children progressing to type 1 diabetes mellitus before the age of four have previously been identified (La Torre et al. 2013), but this study also revealed an association of low lysophosphatidylcholine levels to gestational infections, however, infections with coxsackie virus during pregnancy has previously shown no association to induction of islet autoimmunity in the offspring (Füchtenbusch et al. 2001).

Oxidized phosphatidylcholine levels at 1 month were predictors of an increase in C-peptide levels at 6 and 12 months (Table 2) although this did not reach significance after correction for multiple testing. The coefficient of variation of total oxidized phosphatidylcholine were 43.3% in the quality control samples, and 20.4% in the technical quality control samples indicating low reproducibility of these particular MRMs, so caution should be made when interpreting these results. Oxidation of lipids occurs in both initiation and resolution of inflammation as pro-inflammatory and anti-inflammatory mediators. For instance, eicosanoids, signaling molecules participating in the initiation of the early events in acute inflammation, are derived from oxidation of 20 carbon fatty acids (Lei et al. 2015). In this study a higher level of oxidized phosphatidylcholine at 1 month was predictive of an increase in C-peptide after 6 and 12 months, suggesting that oxidized phosphatidylcholine in this situation may be mediating anti-inflammatory pathways.

A limitation of this study, as with most lipidomics studies, is the inability to identify double bond position in the acyl chains of lipid species. The lipidomic analysis performed in this study was based on scheduled MRM with internal lipid standards for each class of lipids added to the samples with the limitation of not reflecting the point of saturations in the various fatty acid chains in the lipids. While methods to determine acyl chain double bond position do exist these are not compatible with high through put quantification. Another limitation of the study is the lack of lipidomic profiles from healthy controls. Ethically, it is very challenging to justify including healthy children in studies where blood samples are drawn, and we have not been successful in obtaining blood samples from healthy children yet.

## Conclusions

This study provides further evidence for the potential of lipidomic profiles in assessing beta-cell function in type 1 diabetes, and the effects of dysregulated lipid metabolism on the progression of beta-cell destruction. Future studies will aim at investigating prediction and progression models for the development of type 1 diabetes before seroconversion in genetically high-risk children.

## Electronic supplementary material

Below is the link to the electronic supplementary material.

Supplementary material 1 (DOCX 13 KB)

Supplementary material 2 (DOCX 141 KB)

Supplementary material 3 (DOCX 69 KB)

## Abbreviations

dhCer: Dihydroceramide
Cer: Ceramide
MHC: Monohexosylceramide
DHC: Dihexosylceramide
THC: Trihexosylceramide
GM3: GM3 ganglioside
SM: Sphingomyelin
PC: Phosphatidylcholine
OxPC: Oxidized phosphatidylcholine
PC(O): Alkylphosphatidylcholine
PC(P): Plasmalogen, alkenylphosphatidylcholine
LPC: Lysophosphatidylcholine
LPC(O): Lysoplatelet activating factor, lysoalkylphosphatidylcholine
PE: Phosphatidylethanolamine
PE(O): Alkylphosphatidylethanolamine
PE(P): Phosphatidylethanolamine plasmalogen
LPE: Lysophosphatidylethanolamine
PI: Phosphatidylinositol
LPI: Lysophosphatidylinositol
PS: Phosphatidylserine
PG: Phosphatidylglycerol
COH: Free cholesterol
CE: Cholesteryl ester
DG: Diacylglycerol
TG: Triacylglycerol
