# Supplementary material for: Plasma lipid species at type 1 diabetes onset predict residual beta-cell function after 6 months
Source: Metabolomics. 2018 Dec 4;14(12):158. doi: 10.1007/s11306-018-1456-3 (PMC6280838; doi:10.1007/s11306-018-1456-3)
Supplement: Supplementary file 1 — Supplementary material 1 (DOCX 13 KB) [file 11306_2018_1456_MOESM1_ESM.docx]

Supplementary table 1. List of stable isotope and non-physiological lipid standards

| Lipid class | Internal standard | Concentration (pmol/sample) |
| --- | --- | --- |
| Ceramide | Ceramide(17:0) | 100 |
| Dihydroceramide | Dihydroceramide 8:0) | 50 |
| Monohexosylceramide | Glucosylceramide(16:0 d3) | 50 |
| Trihexosylceramide | Trihexosylceramide(17:0) | 50 |
| GM3 ganglioside | Trihexosylceramide(17:0) | 50 |
| Sphingomyelin | Sphingomyelin(12:0) | 200 |
| Phosphatidylcholine | Phosphatidylcholine(13:0/13:0) | 100 |
| Alkylphosphatidylcholine | Phosphatidylcholine(13:0/13:0) | 100 |
| Alkenylphosphatidylcholine | Phosphatidylcholine(13:0/13:0) | 100 |
| Lysophosphatidylcholine | Lysophosphatidylcholine(13:0) | 100 |
| Lysoalkylphosphatidylcholine | Lysophosphatidylcholine(13:0) | 100 |
| Phosphatidylethanolamine | Phosphatidylethanolamine(17:0/17:0) | 100 |
| Alkylphosphatidylethanolamine | Phosphatidylethanolamine(17:0/17:0) | 100 |
| Alkenylphosphatidylethanolamine | Phosphatidylethanolamine(17:0/17:0) | 100 |
| Lysophosphatidylethanolamine | Lysophosphatidylethanolamine(14:0) | 100 |
| Phosphatidylinositol | Phosphatidylethanolamine(17:0/17:0) | 100 |
| Cholesterol | Cholesterol (D7) | 10000 |
| Cholesterol ester | Cholesterol ester(18:0 d6) | 1000 |
| Diacylglycerol | Diacylglycerol(15:0/15:0) | 200 |
| Triacylglycerol | Glyceryl triheptadecanoate(17:0/17:0/17:0) | 100 |
