# Supplementary material for: Plasma lipid species at type 1 diabetes onset predict residual beta-cell function after 6 months
Source: Metabolomics. 2018 Dec 4;14(12):158. doi: 10.1007/s11306-018-1456-3 (PMC6280838; doi:10.1007/s11306-018-1456-3)
Supplement: Supplementary file 2 — Supplementary material 2 (DOCX 141 KB) [file 11306_2018_1456_MOESM2_ESM.docx]

Supplementary table 1 Single lipids association to change in C-peptide levels from 1 to 3, 6 and 12 months.

| Predictor | Change in Outcome * | 95%CI(Lower) | 95%CI(Upper) | p-value | p-value(Benjamini-Hochberg) | Change in Outcome * | 95%CI(Lower) | 95%CI(Upper) | p-value | p-value(Benjamini-Hochberg) | Change in Outcome * | 95%CI(Lower) | 95%CI(Upper) | p-value | p-value(Benjamini-Hochberg) |
| --- | --- | --- | --- | --- | --- | --- | --- | --- | --- | --- | --- | --- | --- | --- | --- |
|  | cpep3_1 |  |  |  |  | cpep6_1 |  |  |  |  | cpep12_1 |  |  |  |  |
| dhCer 16:0 | 2.9 | -35 | 41 | 8.81E-01 | 9.98E-01 | 17.6 | -29 | 65 | 4.63E-01 | 7.90E-01 | -3.1 | -65 | 59 | 9.23E-01 | 9.78E-01 |
| dhCer 18:0 | 4.8 | -42 | 52 | 8.40E-01 | 9.98E-01 | 4.8 | -57 | 66 | 8.78E-01 | 9.55E-01 | -17.4 | -88 | 53 | 6.29E-01 | 8.57E-01 |
| dhCer 20:0 | -9.8 | -55 | 36 | 6.74E-01 | 9.98E-01 | -27.8 | -87 | 31 | 3.57E-01 | 7.19E-01 | -70.7 | -148 | 7 | 7.68E-02 | 3.62E-01 |
| dhCer 22:0 | 25.1 | -20 | 71 | 2.81E-01 | 9.98E-01 | 7.8 | -47 | 63 | 7.83E-01 | 9.27E-01 | -31.3 | -121 | 58 | 4.94E-01 | 7.71E-01 |
| dhCer 24:0 | 17.9 | -30 | 66 | 4.69E-01 | 9.98E-01 | -0.9 | -60 | 58 | 9.77E-01 | 9.88E-01 | -39.4 | -137 | 58 | 4.30E-01 | 7.18E-01 |
| dhCer 24:1 | 4.1 | -28 | 36 | 8.01E-01 | 9.98E-01 | 1.0 | -40 | 42 | 9.61E-01 | 9.86E-01 | -58.3 | -138 | 22 | 1.57E-01 | 4.69E-01 |
| Cer 16:0 | -16.3 | -56 | 23 | 4.20E-01 | 9.98E-01 | -37.5 | -89 | 14 | 1.54E-01 | 4.45E-01 | -30.6 | -92 | 31 | 3.33E-01 | 6.42E-01 |
| Cer 18:0 | -3.7 | -55 | 47 | 8.87E-01 | 9.98E-01 | -39.0 | -106 | 28 | 2.57E-01 | 6.18E-01 | -55.7 | -134 | 23 | 1.66E-01 | 4.85E-01 |
| Cer 20:0 | -8.4 | -61 | 44 | 7.54E-01 | 9.98E-01 | -42.2 | -116 | 32 | 2.66E-01 | 6.27E-01 | -72.1 | -159 | 14 | 1.05E-01 | 3.95E-01 |
| Cer 22:0 | -19.3 | -72 | 34 | 4.77E-01 | 9.98E-01 | -62.9 | -131 | 5 | 7.24E-02 | 3.20E-01 | -105.0 | -192 | -18 | **1.95E-02** | 1.81E-01 |
| Cer 24:0 | -0.4 | -60 | 59 | 9.88E-01 | 9.98E-01 | -63.0 | -141 | 15 | 1.17E-01 | 4.04E-01 | -103.6 | -203 | -4 | **4.46E-02** | 2.67E-01 |
| Cer 24:1 | 8.5 | -44 | 61 | 7.53E-01 | 9.98E-01 | -64.5 | -136 | 7 | 7.93E-02 | 3.46E-01 | -74.8 | -156 | 6 | 7.22E-02 | 3.50E-01 |
| MHC 16:0 | -36.7 | -90 | 17 | 1.80E-01 | 9.98E-01 | -42.4 | -115 | 30 | 2.55E-01 | 6.15E-01 | -33.8 | -118 | 51 | 4.36E-01 | 7.19E-01 |
| MHC 18:0 | -43.9 | -102 | 15 | 1.44E-01 | 9.98E-01 | -64.3 | -143 | 14 | 1.11E-01 | 3.98E-01 | -44.3 | -133 | 44 | 3.27E-01 | 6.35E-01 |
| MHC 20:0 | -41.8 | -103 | 20 | 1.87E-01 | 9.98E-01 | -9.5 | -89 | 70 | 8.16E-01 | 9.43E-01 | -74.7 | -166 | 17 | 1.13E-01 | 4.00E-01 |
| MHC 22:0 | -61.9 | -113 | -10 | **2.01E-02** | 9.98E-01 | -24.3 | -95 | 46 | 5.00E-01 | 8.14E-01 | -53.2 | -127 | 20 | 1.58E-01 | 4.69E-01 |
| MHC 24:0 | -46.7 | -95 | 2 | 6.30E-02 | 9.98E-01 | -15.6 | -76 | 45 | 6.13E-01 | 8.81E-01 | -32.2 | -106 | 42 | 3.95E-01 | 7.01E-01 |
| MHC 24:1 | -33.1 | -77 | 11 | 1.41E-01 | 9.98E-01 | -32.6 | -91 | 26 | 2.78E-01 | 6.38E-01 | -47.7 | -120 | 25 | 2.00E-01 | 5.10E-01 |
| DHC 16:0 | -9.2 | -54 | 36 | 6.87E-01 | 9.98E-01 | -12.5 | -79 | 54 | 7.14E-01 | 9.01E-01 | -58.7 | -127 | 9 | 9.30E-02 | 3.85E-01 |
| DHC 18:0 | -9.7 | -65 | 45 | 7.31E-01 | 9.98E-01 | -8.4 | -83 | 66 | 8.26E-01 | 9.43E-01 | -36.3 | -124 | 52 | 4.21E-01 | 7.17E-01 |
| DHC 20:0 | 34.1 | -12 | 80 | 1.50E-01 | 9.98E-01 | -20.4 | -81 | 40 | 5.10E-01 | 8.19E-01 | -19.9 | -90 | 50 | 5.80E-01 | 8.29E-01 |
| DHC 22:0 | 24.3 | -28 | 77 | 3.65E-01 | 9.98E-01 | -4.4 | -73 | 64 | 9.01E-01 | 9.55E-01 | -44.9 | -126 | 36 | 2.79E-01 | 5.80E-01 |
| DHC 24:0 | 6.8 | -55 | 69 | 8.31E-01 | 9.98E-01 | 1.2 | -82 | 84 | 9.77E-01 | 9.88E-01 | -45.0 | -142 | 52 | 3.68E-01 | 6.80E-01 |
| DHC 24:1 | -30.3 | -87 | 26 | 2.97E-01 | 9.98E-01 | -3.4 | -85 | 78 | 9.34E-01 | 9.70E-01 | -62.2 | -151 | 26 | 1.71E-01 | 4.91E-01 |
| THC 16:0 | -13.8 | -83 | 55 | 6.95E-01 | 9.98E-01 | 7.9 | -78 | 94 | 8.57E-01 | 9.55E-01 | -62.0 | -169 | 44 | 2.56E-01 | 5.71E-01 |
| THC 18:0 | 45.2 | -26 | 116 | 2.16E-01 | 9.98E-01 | 30.1 | -64 | 125 | 5.34E-01 | 8.42E-01 | -5.6 | -113 | 101 | 9.18E-01 | 9.78E-01 |
| THC 20:0 | -18.3 | -75 | 39 | 5.32E-01 | 9.98E-01 | -15.1 | -88 | 58 | 6.85E-01 | 9.01E-01 | -91.5 | -179 | -4 | **4.21E-02** | 2.61E-01 |
| THC 22:0 | -14.9 | -67 | 37 | 5.76E-01 | 9.98E-01 | 12.9 | -55 | 80 | 7.09E-01 | 9.01E-01 | -20.0 | -107 | 67 | 6.53E-01 | 8.65E-01 |
| THC 24:0 | 0.9 | -57 | 59 | 9.76E-01 | 9.98E-01 | 27.5 | -48 | 103 | 4.78E-01 | 8.07E-01 | -9.6 | -104 | 85 | 8.43E-01 | 9.50E-01 |
| THC 24:1 | -0.2 | -62 | 62 | 9.94E-01 | 9.98E-01 | 10.7 | -67 | 88 | 7.88E-01 | 9.27E-01 | -34.7 | -129 | 60 | 4.74E-01 | 7.54E-01 |
| GM3 16:0 | -38.3 | -98 | 22 | 2.15E-01 | 9.98E-01 | -39.0 | -123 | 45 | 3.63E-01 | 7.26E-01 | -76.0 | -174 | 22 | 1.30E-01 | 4.24E-01 |
| GM3 18:0 | -7.2 | -74 | 59 | 8.33E-01 | 9.98E-01 | -5.1 | -92 | 82 | 9.10E-01 | 9.55E-01 | -56.0 | -157 | 45 | 2.80E-01 | 5.80E-01 |
| GM3 20:0 | -4.2 | -62 | 54 | 8.88E-01 | 9.98E-01 | -41.2 | -113 | 30 | 2.63E-01 | 6.22E-01 | -59.1 | -147 | 28 | 1.89E-01 | 5.06E-01 |
| GM3 22:0 | -26.0 | -76 | 24 | 3.09E-01 | 9.98E-01 | -35.9 | -105 | 33 | 3.12E-01 | 6.61E-01 | -73.5 | -153 | 6 | 7.33E-02 | 3.50E-01 |
| GM3 24:0 | -3.7 | -60 | 53 | 8.99E-01 | 9.98E-01 | 36.5 | -34 | 107 | 3.13E-01 | 6.61E-01 | -6.3 | -92 | 79 | 8.86E-01 | 9.59E-01 |
| GM3 24:1 | -18.3 | -71 | 34 | 4.94E-01 | 9.98E-01 | -42.6 | -109 | 23 | 2.09E-01 | 5.47E-01 | -93.3 | -173 | -14 | **2.36E-02** | 2.03E-01 |
| SM 31:1 | 6.4 | -37 | 50 | 7.74E-01 | 9.98E-01 | 3.9 | -56 | 63 | 8.98E-01 | 9.55E-01 | 29.7 | -44 | 103 | 4.33E-01 | 7.18E-01 |
| SM 32:0 | 4.2 | -56 | 64 | 8.92E-01 | 9.98E-01 | 1.5 | -65 | 68 | 9.66E-01 | 9.88E-01 | 37.7 | -68 | 143 | 4.84E-01 | 7.60E-01 |
| SM 32:1 | -2.3 | -66 | 62 | 9.43E-01 | 9.98E-01 | -11.4 | -92 | 70 | 7.83E-01 | 9.27E-01 | 14.5 | -95 | 124 | 7.96E-01 | 9.25E-01 |
| SM 32:2 | 4.8 | -57 | 66 | 8.78E-01 | 9.98E-01 | -65.1 | -142 | 12 | 1.01E-01 | 3.95E-01 | -12.7 | -107 | 81 | 7.92E-01 | 9.25E-01 |
| SM 33:1 | -4.6 | -53 | 43 | 8.51E-01 | 9.98E-01 | 10.4 | -45 | 66 | 7.13E-01 | 9.01E-01 | 2.6 | -85 | 91 | 9.55E-01 | 9.81E-01 |
| SM 34:0 | 0.5 | -41 | 42 | 9.81E-01 | 9.98E-01 | 25.3 | -27 | 78 | 3.49E-01 | 7.11E-01 | -2.4 | -69 | 65 | 9.45E-01 | 9.81E-01 |
| SM 34:1 | -14.3 | -60 | 31 | 5.35E-01 | 9.98E-01 | 15.9 | -44 | 76 | 6.06E-01 | 8.81E-01 | -39.4 | -114 | 35 | 3.02E-01 | 5.98E-01 |
| SM 34:2 | -46.0 | -107 | 15 | 1.43E-01 | 9.98E-01 | -107.8 | -184 | -32 | **6.40E-03** | 7.21E-02 | -119.7 | -205 | -34 | **7.21E-03** | 1.21E-01 |
| SM 34:3 | 0.5 | -62 | 63 | 9.89E-01 | 9.98E-01 | 17.9 | -63 | 99 | 6.65E-01 | 9.01E-01 | 27.8 | -70 | 125 | 5.78E-01 | 8.29E-01 |
| SM 35:1 | -6.8 | -58 | 45 | 7.99E-01 | 9.98E-01 | 15.9 | -47 | 78 | 6.19E-01 | 8.81E-01 | 8.1 | -85 | 101 | 8.65E-01 | 9.59E-01 |
| SM 35:2 | -2.4 | -53 | 48 | 9.26E-01 | 9.98E-01 | 16.1 | -52 | 84 | 6.43E-01 | 8.91E-01 | 13.0 | -68 | 94 | 7.55E-01 | 9.19E-01 |
| SM 36:1 | 8.3 | -42 | 59 | 7.48E-01 | 9.98E-01 | 5.8 | -61 | 73 | 8.65E-01 | 9.55E-01 | -42.7 | -119 | 33 | 2.75E-01 | 5.80E-01 |
| SM 36:2 | -12.1 | -72 | 48 | 6.95E-01 | 9.98E-01 | -45.6 | -127 | 36 | 2.76E-01 | 6.38E-01 | -56.3 | -150 | 38 | 2.43E-01 | 5.68E-01 |
| SM 36:3 | -22.3 | -82 | 38 | 4.68E-01 | 9.98E-01 | 7.0 | -71 | 85 | 8.60E-01 | 9.55E-01 | 9.6 | -81 | 100 | 8.36E-01 | 9.50E-01 |
| SM 37:2 | -24.1 | -79 | 31 | 3.96E-01 | 9.98E-01 | -15.6 | -88 | 57 | 6.72E-01 | 9.01E-01 | 2.5 | -82 | 86 | 9.54E-01 | 9.81E-01 |
| SM 38:1 | 7.9 | -36 | 51 | 7.24E-01 | 9.98E-01 | 25.1 | -30 | 80 | 3.73E-01 | 7.32E-01 | -13.9 | -92 | 64 | 7.28E-01 | 8.98E-01 |
| SM 38:2 | -14.6 | -72 | 42 | 6.17E-01 | 9.98E-01 | -27.8 | -108 | 52 | 4.98E-01 | 8.14E-01 | -85.6 | -172 | 1 | 5.48E-02 | 2.89E-01 |
| SM 39:1 | 9.4 | -32 | 51 | 6.61E-01 | 9.98E-01 | 35.8 | -20 | 92 | 2.14E-01 | 5.54E-01 | 51.2 | -30 | 132 | 2.17E-01 | 5.27E-01 |
| SM 41:1 | 13.7 | -30 | 57 | 5.41E-01 | 9.98E-01 | 33.7 | -21 | 89 | 2.34E-01 | 5.73E-01 | 10.4 | -91 | 112 | 8.41E-01 | 9.50E-01 |
| SM 41:2 | -2.6 | -61 | 55 | 9.31E-01 | 9.98E-01 | 0.2 | -75 | 75 | 9.95E-01 | 9.95E-01 | 0.3 | -100 | 100 | 9.95E-01 | 9.95E-01 |
| SM 42:1 | 14.5 | -19 | 48 | 3.96E-01 | 9.98E-01 | 27.1 | -16 | 71 | 2.23E-01 | 5.57E-01 | -14.0 | -96 | 69 | 7.41E-01 | 9.05E-01 |
| PC 28:0 | -2.2 | -21 | 16 | 8.17E-01 | 9.98E-01 | -23.5 | -77 | 30 | 3.92E-01 | 7.43E-01 | 6.9 | -20 | 34 | 6.20E-01 | 8.48E-01 |
| PC 29:0 | 8.1 | -29 | 46 | 6.74E-01 | 9.98E-01 | 13.3 | -44 | 70 | 6.50E-01 | 8.91E-01 | 43.3 | -19 | 106 | 1.80E-01 | 4.96E-01 |
| PC 30:0 | -8.9 | -60 | 42 | 7.35E-01 | 9.98E-01 | -20.7 | -104 | 62 | 6.26E-01 | 8.81E-01 | 18.9 | -47 | 85 | 5.77E-01 | 8.29E-01 |
| PC 31:0 | 5.3 | -40 | 51 | 8.19E-01 | 9.98E-01 | 17.0 | -39 | 73 | 5.53E-01 | 8.57E-01 | 43.8 | -25 | 113 | 2.18E-01 | 5.27E-01 |
| PC 31:1 | -0.5 | -46 | 45 | 9.82E-01 | 9.98E-01 | 12.7 | -55 | 80 | 7.15E-01 | 9.01E-01 | 9.9 | -84 | 104 | 8.38E-01 | 9.50E-01 |
| PC 32:0 | -23.9 | -74 | 26 | 3.48E-01 | 9.98E-01 | -3.4 | -65 | 58 | 9.14E-01 | 9.55E-01 | -48.3 | -120 | 24 | 1.92E-01 | 5.06E-01 |
| PC 32:1 | -9.9 | -65 | 45 | 7.24E-01 | 9.98E-01 | -83.5 | -158 | -9 | **2.98E-02** | 1.89E-01 | -62.1 | -140 | 16 | 1.22E-01 | 4.20E-01 |
| PC 32:2 | -8.6 | -62 | 44 | 7.51E-01 | 9.98E-01 | -68.4 | -155 | 19 | 1.26E-01 | 4.21E-01 | -8.5 | -91 | 74 | 8.40E-01 | 9.50E-01 |
| PC 32:3 | 8.7 | -46 | 63 | 7.53E-01 | 9.98E-01 | 4.2 | -72 | 81 | 9.14E-01 | 9.55E-01 | 65.0 | -18 | 148 | 1.29E-01 | 4.24E-01 |
| PC 33:0 | -8.0 | -57 | 41 | 7.49E-01 | 9.98E-01 | 12.4 | -46 | 71 | 6.81E-01 | 9.01E-01 | 11.7 | -64 | 87 | 7.61E-01 | 9.23E-01 |
| PC 33:1 | 6.5 | -41 | 54 | 7.87E-01 | 9.98E-01 | -6.8 | -66 | 52 | 8.20E-01 | 9.43E-01 | 24.6 | -44 | 93 | 4.85E-01 | 7.60E-01 |
| PC 33:2 | -0.3 | -61 | 61 | 9.93E-01 | 9.98E-01 | 37.6 | -34 | 109 | 3.07E-01 | 6.61E-01 | 78.6 | -10 | 167 | 8.46E-02 | 3.73E-01 |
| PC 33:3 | 47.4 | -9 | 104 | 1.04E-01 | 9.98E-01 | 20.6 | -52 | 93 | 5.78E-01 | 8.69E-01 | 50.9 | -36 | 138 | 2.53E-01 | 5.69E-01 |
| PC 34:0 | 2.0 | -44 | 48 | 9.32E-01 | 9.98E-01 | 25.1 | -38 | 88 | 4.36E-01 | 7.75E-01 | -34.9 | -106 | 36 | 3.40E-01 | 6.45E-01 |
| PC 34:1 | -19.8 | -81 | 41 | 5.27E-01 | 9.98E-01 | -65.0 | -149 | 19 | 1.33E-01 | 4.31E-01 | -44.3 | -141 | 52 | 3.72E-01 | 6.80E-01 |
| PC 34:2 | -20.3 | -76 | 36 | 4.79E-01 | 9.98E-01 | -0.2 | -74 | 73 | 9.95E-01 | 9.95E-01 | 11.7 | -75 | 99 | 7.93E-01 | 9.25E-01 |
| PC 34:3 | 21.9 | -39 | 83 | 4.85E-01 | 9.98E-01 | -17.2 | -98 | 64 | 6.79E-01 | 9.01E-01 | 3.0 | -92 | 98 | 9.50E-01 | 9.81E-01 |
| PC 34:4 | 36.3 | -22 | 95 | 2.27E-01 | 9.98E-01 | -4.8 | -82 | 72 | 9.02E-01 | 9.55E-01 | 53.8 | -28 | 136 | 2.02E-01 | 5.13E-01 |
| PC 34:5 | 16.4 | -26 | 59 | 4.49E-01 | 9.98E-01 | 1.4 | -54 | 57 | 9.59E-01 | 9.86E-01 | 22.2 | -37 | 82 | 4.66E-01 | 7.51E-01 |
| PC 35:0 | 1.7 | -44 | 47 | 9.43E-01 | 9.98E-01 | 21.4 | -36 | 79 | 4.63E-01 | 7.90E-01 | 17.9 | -59 | 95 | 6.49E-01 | 8.65E-01 |
| PC 35:1 | 4.2 | -36 | 44 | 8.36E-01 | 9.98E-01 | 11.0 | -42 | 64 | 6.85E-01 | 9.01E-01 | 15.4 | -55 | 85 | 6.67E-01 | 8.65E-01 |
| PC 35:2 | -2.8 | -53 | 47 | 9.12E-01 | 9.98E-01 | 42.8 | -20 | 105 | 1.81E-01 | 5.04E-01 | 42.6 | -37 | 122 | 2.95E-01 | 5.92E-01 |
| PC 35:3 | 38.0 | -15 | 91 | 1.60E-01 | 9.98E-01 | 72.3 | -3 | 148 | 6.41E-02 | 3.10E-01 | 91.3 | 8 | 174 | **3.37E-02** | 2.34E-01 |
| PC 35:4 | 40.4 | -19 | 99 | 1.82E-01 | 9.98E-01 | 67.7 | -14 | 150 | 1.09E-01 | 3.98E-01 | 52.7 | -41 | 146 | 2.72E-01 | 5.80E-01 |
| PC 36:0 | -4.3 | -56 | 47 | 8.68E-01 | 9.98E-01 | -6.6 | -76 | 62 | 8.52E-01 | 9.55E-01 | -92.6 | -175 | -10 | **2.97E-02** | 2.18E-01 |
| PC 36:1 | 14.8 | -40 | 70 | 6.01E-01 | 9.98E-01 | -5.8 | -75 | 63 | 8.69E-01 | 9.55E-01 | -7.3 | -93 | 78 | 8.68E-01 | 9.59E-01 |
| PC 36:2 | 2.1 | -50 | 54 | 9.37E-01 | 9.98E-01 | 54.1 | -17 | 126 | 1.42E-01 | 4.43E-01 | 50.2 | -31 | 131 | 2.26E-01 | 5.43E-01 |
| PC 36:3 | 51.8 | -10 | 114 | 1.06E-01 | 9.98E-01 | 91.0 | 10 | 172 | **3.00E-02** | 1.89E-01 | 96.4 | 0 | 192 | 5.17E-02 | 2.78E-01 |
| PC 36:4a | 16.4 | -46 | 78 | 6.05E-01 | 9.98E-01 | 84.1 | 11 | 157 | **2.62E-02** | 1.72E-01 | 118.2 | 28 | 208 | **1.14E-02** | 1.59E-01 |
| PC 36:4b | 15.9 | -32 | 64 | 5.19E-01 | 9.98E-01 | 3.7 | -56 | 63 | 9.04E-01 | 9.55E-01 | -18.3 | -94 | 58 | 6.39E-01 | 8.61E-01 |
| PC 36:5 | 14.6 | -28 | 57 | 5.00E-01 | 9.98E-01 | -3.0 | -57 | 51 | 9.13E-01 | 9.55E-01 | 4.3 | -60 | 69 | 8.97E-01 | 9.63E-01 |
| PC 36:6 | 2.9 | -48 | 54 | 9.10E-01 | 9.98E-01 | -26.0 | -94 | 42 | 4.57E-01 | 7.87E-01 | 5.2 | -70 | 80 | 8.92E-01 | 9.61E-01 |
| PC 37:4 | -1.2 | -50 | 48 | 9.63E-01 | 9.98E-01 | 24.7 | -36 | 86 | 4.30E-01 | 7.75E-01 | 15.3 | -68 | 98 | 7.18E-01 | 8.96E-01 |
| PC 37:5 | 6.1 | -37 | 49 | 7.84E-01 | 9.98E-01 | -3.4 | -60 | 53 | 9.06E-01 | 9.55E-01 | 8.6 | -57 | 74 | 7.96E-01 | 9.25E-01 |
| PC 37:6 | -22.5 | -77 | 32 | 4.18E-01 | 9.98E-01 | -20.0 | -89 | 49 | 5.69E-01 | 8.66E-01 | -8.9 | -92 | 74 | 8.33E-01 | 9.50E-01 |
| PC 38:2 | 18.3 | -26 | 62 | 4.18E-01 | 9.98E-01 | 51.8 | -6 | 110 | 8.23E-02 | 3.50E-01 | 3.9 | -71 | 79 | 9.19E-01 | 9.78E-01 |
| PC 38:3 | 52.6 | -2 | 108 | 6.39E-02 | 9.98E-01 | 2.6 | -72 | 77 | 9.46E-01 | 9.77E-01 | -10.2 | -94 | 74 | 8.12E-01 | 9.40E-01 |
| PC 38:4 | 26.2 | -22 | 74 | 2.89E-01 | 9.98E-01 | 19.9 | -39 | 79 | 5.10E-01 | 8.19E-01 | -14.8 | -90 | 60 | 7.01E-01 | 8.90E-01 |
| PC 38:5 | 17.8 | -49 | 85 | 6.03E-01 | 9.98E-01 | 15.5 | -65 | 96 | 7.06E-01 | 9.01E-01 | 0.4 | -100 | 101 | 9.94E-01 | 9.95E-01 |
| PC 38:6a | 61.7 | 9 | 114 | **2.29E-02** | 9.98E-01 | 95.7 | 27 | 164 | **7.12E-03** | 7.62E-02 | 96.0 | 17 | 175 | **1.95E-02** | 1.81E-01 |
| PC 38:6b | -39.6 | -95 | 16 | 1.66E-01 | 9.98E-01 | -48.9 | -121 | 23 | 1.87E-01 | 5.05E-01 | -52.1 | -137 | 32 | 2.30E-01 | 5.49E-01 |
| PC 38:7 | 11.2 | -35 | 57 | 6.34E-01 | 9.98E-01 | 6.6 | -55 | 69 | 8.34E-01 | 9.43E-01 | 14.5 | -65 | 94 | 7.22E-01 | 8.97E-01 |
| PC 39:5 | -2.0 | -63 | 59 | 9.49E-01 | 9.98E-01 | 9.1 | -73 | 91 | 8.29E-01 | 9.43E-01 | 7.5 | -87 | 102 | 8.77E-01 | 9.59E-01 |
| PC 39:6 | -22.7 | -75 | 30 | 3.96E-01 | 9.98E-01 | -16.7 | -85 | 51 | 6.31E-01 | 8.83E-01 | -14.4 | -91 | 62 | 7.14E-01 | 8.96E-01 |
| PC 39:7 | -1.8 | -40 | 36 | 9.25E-01 | 9.98E-01 | -4.9 | -51 | 42 | 8.36E-01 | 9.43E-01 | -5.0 | -60 | 50 | 8.58E-01 | 9.56E-01 |
| PC 40:4 | 30.1 | -23 | 83 | 2.71E-01 | 9.98E-01 | -7.8 | -77 | 61 | 8.24E-01 | 9.43E-01 | -23.4 | -107 | 61 | 5.86E-01 | 8.35E-01 |
| PC 40:5 | 7.4 | -60 | 74 | 8.29E-01 | 9.98E-01 | -38.9 | -127 | 49 | 3.86E-01 | 7.41E-01 | -45.7 | -151 | 60 | 3.97E-01 | 7.01E-01 |
| PC 40:6 | -23.6 | -78 | 31 | 4.03E-01 | 9.98E-01 | -57.7 | -129 | 13 | 1.15E-01 | 4.04E-01 | -70.6 | -153 | 11 | 9.47E-02 | 3.85E-01 |
| PC 40:7 | -21.2 | -73 | 31 | 4.24E-01 | 9.98E-01 | 7.6 | -63 | 78 | 8.33E-01 | 9.43E-01 | 14.8 | -69 | 99 | 7.29E-01 | 8.98E-01 |
| PC 40:8 | 9.3 | -44 | 63 | 7.36E-01 | 9.98E-01 | 44.9 | -21 | 111 | 1.85E-01 | 5.05E-01 | 52.2 | -28 | 133 | 2.08E-01 | 5.20E-01 |
| PC(O-32:0) | -18.9 | -70 | 32 | 4.66E-01 | 9.98E-01 | -4.4 | -70 | 61 | 8.94E-01 | 9.55E-01 | -61.1 | -143 | 21 | 1.47E-01 | 4.63E-01 |
| PC(O-32:1) | -15.4 | -74 | 43 | 6.09E-01 | 9.98E-01 | 10.7 | -66 | 87 | 7.83E-01 | 9.27E-01 | -17.6 | -110 | 74 | 7.08E-01 | 8.96E-01 |
| PC(O-32:2) | 17.0 | -38 | 72 | 5.45E-01 | 9.98E-01 | 45.5 | -16 | 108 | 1.53E-01 | 4.45E-01 | 29.8 | -40 | 100 | 4.07E-01 | 7.01E-01 |
| PC(O-34:1) | -16.3 | -74 | 41 | 5.79E-01 | 9.98E-01 | 15.0 | -59 | 89 | 6.92E-01 | 9.01E-01 | -8.4 | -96 | 79 | 8.51E-01 | 9.54E-01 |
| PC(O-34:2) | 45.8 | -3 | 94 | 6.79E-02 | 9.98E-01 | 67.7 | 1 | 134 | **4.88E-02** | 2.65E-01 | 86.6 | 3 | 170 | **4.47E-02** | 2.67E-01 |
| PC(O-34:3) | 50.0 | -13 | 113 | 1.20E-01 | 9.98E-01 | 56.9 | -21 | 134 | 1.54E-01 | 4.45E-01 | 54.7 | -38 | 147 | 2.49E-01 | 5.69E-01 |
| PC(O-34:4) | 25.6 | -22 | 73 | 2.91E-01 | 9.98E-01 | 19.9 | -28 | 67 | 4.12E-01 | 7.58E-01 | 31.9 | -22 | 86 | 2.47E-01 | 5.69E-01 |
| PC(O-36:0) | 2.7 | -45 | 50 | 9.12E-01 | 9.98E-01 | 0.6 | -63 | 64 | 9.85E-01 | 9.91E-01 | -88.1 | -169 | -7 | **3.49E-02** | 2.35E-01 |
| PC(O-36:1) | 10.1 | -43 | 63 | 7.09E-01 | 9.98E-01 | 17.8 | -55 | 91 | 6.33E-01 | 8.83E-01 | -5.9 | -87 | 75 | 8.86E-01 | 9.59E-01 |
| PC(O-36:2) | -5.3 | -60 | 50 | 8.51E-01 | 9.98E-01 | 29.7 | -44 | 103 | 4.31E-01 | 7.75E-01 | 32.0 | -61 | 125 | 5.01E-01 | 7.71E-01 |
| PC(O-36:3) | 22.9 | -30 | 76 | 3.99E-01 | 9.98E-01 | 38.7 | -33 | 111 | 2.95E-01 | 6.51E-01 | 56.1 | -32 | 145 | 2.17E-01 | 5.27E-01 |
| PC(O-36:4) | 27.8 | -27 | 82 | 3.22E-01 | 9.98E-01 | 47.1 | -17 | 111 | 1.54E-01 | 4.45E-01 | 58.5 | -18 | 135 | 1.35E-01 | 4.31E-01 |
| PC(O-36:5) | 15.4 | -30 | 61 | 5.08E-01 | 9.98E-01 | 14.2 | -39 | 68 | 6.04E-01 | 8.81E-01 | 27.3 | -37 | 91 | 4.06E-01 | 7.01E-01 |
| PC(O-38:4) | 24.5 | -41 | 90 | 4.65E-01 | 9.98E-01 | 17.1 | -73 | 107 | 7.10E-01 | 9.01E-01 | -3.0 | -97 | 91 | 9.50E-01 | 9.81E-01 |
| PC(O-38:5) | 14.0 | -53 | 81 | 6.82E-01 | 9.98E-01 | 45.3 | -37 | 127 | 2.83E-01 | 6.44E-01 | 13.1 | -84 | 111 | 7.92E-01 | 9.25E-01 |
| PC(O-40:5) | -26.1 | -86 | 34 | 3.99E-01 | 9.98E-01 | -30.3 | -116 | 56 | 4.91E-01 | 8.14E-01 | -56.6 | -159 | 46 | 2.81E-01 | 5.80E-01 |
| PC(O-40:6) | -12.2 | -61 | 37 | 6.24E-01 | 9.98E-01 | -22.2 | -86 | 42 | 5.00E-01 | 8.14E-01 | -18.5 | -101 | 64 | 6.61E-01 | 8.65E-01 |
| PC(O-40:7) | -21.0 | -73 | 31 | 4.27E-01 | 9.98E-01 | -6.9 | -73 | 59 | 8.39E-01 | 9.44E-01 | 10.8 | -63 | 85 | 7.76E-01 | 9.25E-01 |
| PC(P-30:0) | 6.7 | -46 | 59 | 8.02E-01 | 9.98E-01 | 41.6 | -24 | 107 | 2.19E-01 | 5.54E-01 | 36.0 | -43 | 115 | 3.73E-01 | 6.80E-01 |
| PC(P-32:0) | -9.4 | -68 | 49 | 7.55E-01 | 9.98E-01 | 35.4 | -46 | 116 | 3.94E-01 | 7.43E-01 | -40.8 | -134 | 52 | 3.93E-01 | 7.01E-01 |
| PC(P-32:1) | 24.6 | -26 | 75 | 3.40E-01 | 9.98E-01 | 49.3 | -15 | 113 | 1.35E-01 | 4.31E-01 | 29.8 | -57 | 116 | 5.02E-01 | 7.71E-01 |
| PC(P-34:1) | 49.5 | 0 | 99 | 5.31E-02 | 9.98E-01 | 63.7 | 0 | 127 | 5.11E-02 | 2.65E-01 | 91.0 | 7 | 175 | **3.63E-02** | 2.35E-01 |
| PC(P-34:2) | 31.1 | -25 | 88 | 2.83E-01 | 9.98E-01 | 44.1 | -27 | 115 | 2.27E-01 | 5.60E-01 | 16.4 | -73 | 105 | 7.19E-01 | 8.96E-01 |
| PC(P-34:3) | 27.6 | -14 | 69 | 1.93E-01 | 9.98E-01 | 6.0 | -46 | 58 | 8.23E-01 | 9.43E-01 | -21.1 | -85 | 42 | 5.15E-01 | 7.81E-01 |
| PC(P-36:2) | 29.8 | -29 | 88 | 3.20E-01 | 9.98E-01 | 24.3 | -50 | 99 | 5.22E-01 | 8.31E-01 | 20.9 | -70 | 112 | 6.54E-01 | 8.65E-01 |
| PC(P-36:4) | 32.8 | -28 | 94 | 2.93E-01 | 9.98E-01 | 13.6 | -69 | 96 | 7.47E-01 | 9.13E-01 | -7.2 | -93 | 79 | 8.70E-01 | 9.59E-01 |
| PC(P-36:5) | 9.9 | -39 | 59 | 6.92E-01 | 9.98E-01 | -9.6 | -76 | 57 | 7.79E-01 | 9.27E-01 | -16.0 | -93 | 61 | 6.87E-01 | 8.78E-01 |
| PC(P-38:4) | 12.2 | -53 | 77 | 7.14E-01 | 9.98E-01 | 4.2 | -74 | 82 | 9.15E-01 | 9.55E-01 | -28.2 | -126 | 70 | 5.75E-01 | 8.29E-01 |
| PC(P-38:5) | 9.3 | -39 | 58 | 7.07E-01 | 9.98E-01 | 14.8 | -48 | 78 | 6.46E-01 | 8.91E-01 | 16.2 | -51 | 83 | 6.37E-01 | 8.61E-01 |
| PC(P-38:6) | -17.2 | -69 | 34 | 5.15E-01 | 9.98E-01 | -32.1 | -106 | 41 | 3.93E-01 | 7.43E-01 | -28.6 | -108 | 51 | 4.84E-01 | 7.60E-01 |
| PC(P-40:6) | -7.3 | -61 | 46 | 7.90E-01 | 9.98E-01 | -9.7 | -84 | 64 | 7.97E-01 | 9.28E-01 | -4.0 | -83 | 75 | 9.21E-01 | 9.78E-01 |
| LPC 14:0 | -2.6 | -51 | 45 | 9.16E-01 | 9.98E-01 | -23.9 | -108 | 60 | 5.79E-01 | 8.69E-01 | 25.5 | -49 | 99 | 5.01E-01 | 7.71E-01 |
| LPC 15:0 | -12.2 | -57 | 32 | 5.93E-01 | 9.98E-01 | 11.0 | -45 | 67 | 7.00E-01 | 9.01E-01 | 19.4 | -44 | 83 | 5.52E-01 | 8.15E-01 |
| LPC 16:0 | -57.5 | -113 | -2 | **4.40E-02** | 9.98E-01 | -33.8 | -108 | 41 | 3.75E-01 | 7.32E-01 | -73.5 | -162 | 15 | 1.08E-01 | 3.95E-01 |
| LPC 16:1 | -17.4 | -84 | 49 | 6.09E-01 | 9.98E-01 | -27.0 | -117 | 63 | 5.57E-01 | 8.59E-01 | -43.5 | -151 | 64 | 4.28E-01 | 7.18E-01 |
| LPC 17:0 | -24.5 | -74 | 25 | 3.38E-01 | 9.98E-01 | 5.6 | -60 | 71 | 8.67E-01 | 9.55E-01 | 1.8 | -72 | 76 | 9.61E-01 | 9.83E-01 |
| LPC 17:1 | 4.0 | -44 | 52 | 8.69E-01 | 9.98E-01 | 25.9 | -39 | 90 | 4.33E-01 | 7.75E-01 | 29.8 | -48 | 107 | 4.53E-01 | 7.37E-01 |
| LPC 18:0 | -33.2 | -85 | 18 | 2.08E-01 | 9.98E-01 | -10.9 | -77 | 56 | 7.48E-01 | 9.13E-01 | -42.5 | -122 | 37 | 2.95E-01 | 5.92E-01 |
| LPC 18:1 | -6.5 | -58 | 45 | 8.05E-01 | 9.98E-01 | 43.3 | -24 | 110 | 2.07E-01 | 5.47E-01 | 32.3 | -44 | 108 | 4.07E-01 | 7.01E-01 |
| LPC 18:2 | 3.9 | -45 | 53 | 8.76E-01 | 9.98E-01 | 76.0 | 16 | 136 | **1.40E-02** | 1.10E-01 | 99.5 | 15 | 184 | **2.31E-02** | 2.03E-01 |
| LPC 18:3 | 42.2 | -7 | 92 | 9.70E-02 | 9.98E-01 | 50.4 | -14 | 114 | 1.26E-01 | 4.21E-01 | 73.4 | -8 | 154 | 7.91E-02 | 3.67E-01 |
| LPC 20:0 | -27.0 | -70 | 16 | 2.26E-01 | 9.98E-01 | 17.9 | -34 | 70 | 5.03E-01 | 8.14E-01 | -39.9 | -112 | 32 | 2.82E-01 | 5.80E-01 |
| LPC 20:1 | -4.9 | -45 | 35 | 8.13E-01 | 9.98E-01 | 19.7 | -30 | 70 | 4.43E-01 | 7.75E-01 | 0.6 | -66 | 67 | 9.86E-01 | 9.94E-01 |
| LPC 20:2 | 16.0 | -38 | 70 | 5.64E-01 | 9.98E-01 | 56.0 | -13 | 125 | 1.17E-01 | 4.04E-01 | 81.1 | -1 | 163 | 5.59E-02 | 2.90E-01 |
| LPC 20:3 | 46.3 | -13 | 106 | 1.30E-01 | 9.98E-01 | 73.9 | -13 | 161 | 9.93E-02 | 3.95E-01 | 72.3 | -31 | 176 | 1.74E-01 | 4.92E-01 |
| LPC 20:4 | 19.3 | -37 | 76 | 5.04E-01 | 9.98E-01 | 72.7 | -4 | 150 | 6.76E-02 | 3.13E-01 | 25.1 | -66 | 116 | 5.90E-01 | 8.35E-01 |
| LPC 20:5 | 7.8 | -35 | 51 | 7.25E-01 | 9.98E-01 | 8.5 | -48 | 65 | 7.67E-01 | 9.27E-01 | 5.3 | -63 | 73 | 8.79E-01 | 9.59E-01 |
| LPC 22:0 | -16.8 | -44 | 10 | 2.22E-01 | 9.98E-01 | 14.9 | -20 | 50 | 4.07E-01 | 7.55E-01 | -16.1 | -66 | 34 | 5.32E-01 | 7.98E-01 |
| LPC 22:1 | -0.3 | -18 | 18 | 9.76E-01 | 9.98E-01 | 3.0 | -14 | 20 | 7.29E-01 | 9.10E-01 | 5.1 | -14 | 24 | 5.96E-01 | 8.35E-01 |
| LPC 22:5 | -4.4 | -57 | 48 | 8.69E-01 | 9.98E-01 | 20.0 | -50 | 90 | 5.75E-01 | 8.69E-01 | 14.5 | -62 | 91 | 7.11E-01 | 8.96E-01 |
| LPC 22:6 | -30.4 | -87 | 26 | 2.95E-01 | 9.98E-01 | -12.8 | -92 | 66 | 7.50E-01 | 9.13E-01 | -27.8 | -117 | 61 | 5.41E-01 | 8.06E-01 |
| LPC 24:0 | 0.9 | -34 | 36 | 9.59E-01 | 9.98E-01 | 37.3 | -8 | 83 | 1.08E-01 | 3.98E-01 | 26.8 | -42 | 96 | 4.48E-01 | 7.32E-01 |
| LPC 26:0 | -2.0 | -45 | 41 | 9.28E-01 | 9.98E-01 | 28.4 | -28 | 85 | 3.23E-01 | 6.71E-01 | 15.9 | -55 | 87 | 6.62E-01 | 8.65E-01 |
| LPC(O-16:0) | -27.9 | -88 | 32 | 3.66E-01 | 9.98E-01 | -17.7 | -101 | 65 | 6.77E-01 | 9.01E-01 | -43.8 | -146 | 58 | 4.03E-01 | 7.01E-01 |
| LPC(O-18:0) | -20.6 | -78 | 37 | 4.85E-01 | 9.98E-01 | -29.9 | -107 | 47 | 4.50E-01 | 7.80E-01 | -64.5 | -158 | 29 | 1.78E-01 | 4.95E-01 |
| LPC(O-18:1) | -34.0 | -86 | 18 | 2.02E-01 | 9.98E-01 | -13.3 | -83 | 56 | 7.08E-01 | 9.01E-01 | -67.4 | -148 | 14 | 1.06E-01 | 3.95E-01 |
| LPC(O-20:1) | -23.2 | -79 | 32 | 4.13E-01 | 9.98E-01 | 15.3 | -54 | 85 | 6.67E-01 | 9.01E-01 | 3.2 | -80 | 86 | 9.39E-01 | 9.81E-01 |
| LPC(O-22:0) | -29.5 | -80 | 21 | 2.54E-01 | 9.98E-01 | -17.5 | -85 | 50 | 6.12E-01 | 8.81E-01 | -93.2 | -182 | -5 | **4.13E-02** | 2.61E-01 |
| LPC(O-22:1) | -41.4 | -90 | 8 | 1.01E-01 | 9.98E-01 | -33.1 | -94 | 28 | 2.91E-01 | 6.45E-01 | -83.7 | -159 | -8 | **3.18E-02** | 2.29E-01 |
| LPC(O-24:0) | -10.2 | -61 | 40 | 6.92E-01 | 9.98E-01 | -34.7 | -100 | 31 | 3.01E-01 | 6.52E-01 | -57.3 | -141 | 26 | 1.83E-01 | 5.01E-01 |
| LPC(O-24:1) | -43.4 | -96 | 9 | 1.05E-01 | 9.98E-01 | -53.3 | -121 | 14 | 1.26E-01 | 4.21E-01 | -65.1 | -144 | 13 | 1.07E-01 | 3.95E-01 |
| LPC(O-24:2) | -27.4 | -73 | 18 | 2.38E-01 | 9.98E-01 | -9.8 | -68 | 49 | 7.44E-01 | 9.13E-01 | -30.7 | -98 | 36 | 3.72E-01 | 6.80E-01 |
| PE 32:0 | -13.2 | -69 | 43 | 6.46E-01 | 9.98E-01 | -5.4 | -83 | 72 | 8.91E-01 | 9.55E-01 | -29.4 | -124 | 65 | 5.44E-01 | 8.06E-01 |
| PE 32:1 | 2.6 | -55 | 60 | 9.28E-01 | 9.98E-01 | -54.4 | -128 | 19 | 1.51E-01 | 4.45E-01 | -19.4 | -108 | 69 | 6.68E-01 | 8.65E-01 |
| PE 34:1 | 2.4 | -46 | 51 | 9.23E-01 | 9.98E-01 | -19.1 | -87 | 49 | 5.83E-01 | 8.72E-01 | -22.9 | -102 | 56 | 5.72E-01 | 8.29E-01 |
| PE 34:2 | -8.9 | -67 | 49 | 7.65E-01 | 9.98E-01 | -51.6 | -133 | 30 | 2.20E-01 | 5.54E-01 | 10.9 | -88 | 110 | 8.28E-01 | 9.50E-01 |
| PE 34:3 | 6.6 | -40 | 53 | 7.83E-01 | 9.98E-01 | -23.5 | -96 | 49 | 5.25E-01 | 8.31E-01 | 2.3 | -79 | 83 | 9.56E-01 | 9.81E-01 |
| PE 35:1 | -10.8 | -64 | 43 | 6.94E-01 | 9.98E-01 | -6.4 | -79 | 66 | 8.63E-01 | 9.55E-01 | -11.5 | -91 | 68 | 7.78E-01 | 9.25E-01 |
| PE 35:2 | 19.1 | -42 | 80 | 5.40E-01 | 9.98E-01 | 23.6 | -57 | 104 | 5.65E-01 | 8.64E-01 | 63.4 | -30 | 157 | 1.86E-01 | 5.05E-01 |
| PE 36:0 | -40.5 | -100 | 19 | 1.87E-01 | 9.98E-01 | -37.7 | -116 | 41 | 3.49E-01 | 7.11E-01 | -28.0 | -121 | 65 | 5.57E-01 | 8.18E-01 |
| PE 36:1 | 10.0 | -36 | 56 | 6.67E-01 | 9.98E-01 | -3.8 | -62 | 54 | 8.99E-01 | 9.55E-01 | -5.2 | -73 | 63 | 8.81E-01 | 9.59E-01 |
| PE 36:2 | -4.1 | -50 | 42 | 8.64E-01 | 9.98E-01 | -25.7 | -91 | 40 | 4.43E-01 | 7.75E-01 | 2.5 | -72 | 77 | 9.48E-01 | 9.81E-01 |
| PE 36:3 | -0.2 | -44 | 44 | 9.93E-01 | 9.98E-01 | 0.7 | -64 | 66 | 9.82E-01 | 9.91E-01 | 50.4 | -29 | 129 | 2.14E-01 | 5.27E-01 |
| PE 36:4 | -9.0 | -67 | 49 | 7.64E-01 | 9.98E-01 | -45.3 | -118 | 27 | 2.24E-01 | 5.57E-01 | -51.5 | -146 | 43 | 2.87E-01 | 5.85E-01 |
| PE 36:5 | 18.7 | -36 | 74 | 5.08E-01 | 9.98E-01 | -24.2 | -98 | 50 | 5.22E-01 | 8.31E-01 | 15.1 | -74 | 104 | 7.39E-01 | 9.05E-01 |
| PE 38:3 | 2.4 | -59 | 64 | 9.39E-01 | 9.98E-01 | -61.4 | -147 | 25 | 1.65E-01 | 4.68E-01 | -50.5 | -149 | 48 | 3.19E-01 | 6.28E-01 |
| PE 38:4 | 6.4 | -44 | 57 | 8.04E-01 | 9.98E-01 | -31.2 | -101 | 39 | 3.85E-01 | 7.41E-01 | -41.4 | -119 | 36 | 3.00E-01 | 5.98E-01 |
| PE 38:5 | -6.4 | -58 | 45 | 8.09E-01 | 9.98E-01 | -31.9 | -112 | 48 | 4.38E-01 | 7.75E-01 | -8.4 | -98 | 81 | 8.54E-01 | 9.54E-01 |
| PE 38:6 | -10.6 | -60 | 39 | 6.75E-01 | 9.98E-01 | -41.8 | -108 | 24 | 2.19E-01 | 5.54E-01 | -27.0 | -105 | 51 | 4.97E-01 | 7.71E-01 |
| PE 40:4 | 8.9 | -42 | 59 | 7.30E-01 | 9.98E-01 | -33.9 | -109 | 41 | 3.80E-01 | 7.37E-01 | -39.3 | -117 | 39 | 3.25E-01 | 6.35E-01 |
| PE 40:5 | -2.3 | -48 | 43 | 9.22E-01 | 9.98E-01 | -65.2 | -133 | 3 | 6.27E-02 | 3.10E-01 | -49.3 | -123 | 24 | 1.92E-01 | 5.06E-01 |
| PE 40:6 | -14.9 | -70 | 41 | 6.02E-01 | 9.98E-01 | -77.2 | -147 | -7 | **3.28E-02** | 1.96E-01 | -72.3 | -157 | 12 | 9.67E-02 | 3.88E-01 |
| PE 40:7 | -7.5 | -68 | 53 | 8.09E-01 | 9.98E-01 | -24.8 | -107 | 57 | 5.54E-01 | 8.57E-01 | -4.3 | -100 | 91 | 9.30E-01 | 9.81E-01 |
| PE(O-34:1) | 36.7 | -25 | 98 | 2.47E-01 | 9.98E-01 | 50.2 | -24 | 124 | 1.88E-01 | 5.05E-01 | 113.9 | 25 | 203 | **1.38E-02** | 1.59E-01 |
| PE(O-34:2) | 19.6 | -39 | 78 | 5.14E-01 | 9.98E-01 | 19.7 | -58 | 97 | 6.19E-01 | 8.81E-01 | 77.6 | -12 | 167 | 9.36E-02 | 3.85E-01 |
| PE(O-36:2) | -10.0 | -67 | 47 | 7.33E-01 | 9.98E-01 | -11.5 | -82 | 59 | 7.50E-01 | 9.13E-01 | 79.3 | -9 | 168 | 8.16E-02 | 3.67E-01 |
| PE(O-36:3) | 32.6 | -22 | 87 | 2.47E-01 | 9.98E-01 | 24.8 | -44 | 94 | 4.81E-01 | 8.09E-01 | 92.6 | 14 | 171 | **2.26E-02** | 2.03E-01 |
| PE(O-36:4) | 24.8 | -37 | 86 | 4.30E-01 | 9.98E-01 | 28.9 | -44 | 102 | 4.42E-01 | 7.75E-01 | 98.0 | 12 | 184 | **2.80E-02** | 2.18E-01 |
| PE(O-36:5) | 9.7 | -39 | 58 | 6.98E-01 | 9.98E-01 | 8.8 | -45 | 62 | 7.47E-01 | 9.13E-01 | 38.1 | -27 | 103 | 2.53E-01 | 5.69E-01 |
| PE(O-36:6) | 13.0 | -21 | 47 | 4.60E-01 | 9.98E-01 | 10.2 | -33 | 54 | 6.46E-01 | 8.91E-01 | 9.8 | -32 | 51 | 6.44E-01 | 8.65E-01 |
| PE(O-38:4) | 2.0 | -37 | 41 | 9.22E-01 | 9.98E-01 | -8.7 | -59 | 41 | 7.35E-01 | 9.13E-01 | 69.7 | 2 | 138 | **4.66E-02** | 2.70E-01 |
| PE(O-38:5) | 27.9 | -27 | 83 | 3.25E-01 | 9.98E-01 | 23.3 | -44 | 91 | 4.99E-01 | 8.14E-01 | 97.2 | 18 | 176 | **1.76E-02** | 1.81E-01 |
| PE(O-40:5) | 1.7 | -50 | 53 | 9.48E-01 | 9.98E-01 | -13.9 | -80 | 52 | 6.83E-01 | 9.01E-01 | 60.1 | -23 | 143 | 1.58E-01 | 4.69E-01 |
| PE(O-40:6) | 2.2 | -51 | 56 | 9.36E-01 | 9.98E-01 | -10.9 | -79 | 57 | 7.53E-01 | 9.13E-01 | -2.4 | -86 | 81 | 9.55E-01 | 9.81E-01 |
| PE(O-40:7) | -0.2 | -47 | 47 | 9.95E-01 | 9.98E-01 | -1.1 | -58 | 55 | 9.68E-01 | 9.88E-01 | 51.5 | -11 | 114 | 1.12E-01 | 4.00E-01 |
| PE(P-16:0/18:1) FA | 24.3 | -36 | 85 | 4.32E-01 | 9.98E-01 | 11.6 | -73 | 96 | 7.88E-01 | 9.27E-01 | 25.1 | -70 | 120 | 6.06E-01 | 8.35E-01 |
| PE(P-16:0/18:2) FA | -0.4 | -56 | 55 | 9.88E-01 | 9.98E-01 | -8.9 | -83 | 65 | 8.13E-01 | 9.43E-01 | -0.9 | -91 | 90 | 9.85E-01 | 9.94E-01 |
| PE(P-16:0/20:4) FA | 25.6 | -28 | 79 | 3.48E-01 | 9.98E-01 | 4.1 | -59 | 68 | 8.99E-01 | 9.55E-01 | 28.6 | -57 | 114 | 5.13E-01 | 7.81E-01 |
| PE(P-16:0/22:5) FA | 49.1 | -10 | 108 | 1.04E-01 | 9.98E-01 | 41.5 | -30 | 113 | 2.60E-01 | 6.21E-01 | 65.3 | -24 | 155 | 1.57E-01 | 4.69E-01 |
| PE(P-16:0/22:6) FA | -7.7 | -59 | 44 | 7.72E-01 | 9.98E-01 | -35.5 | -99 | 28 | 2.75E-01 | 6.38E-01 | 1.6 | -75 | 78 | 9.68E-01 | 9.88E-01 |
| PE(P-18:0/18:1) FA | 12.9 | -51 | 77 | 6.92E-01 | 9.98E-01 | -5.0 | -84 | 74 | 9.01E-01 | 9.55E-01 | 13.4 | -82 | 109 | 7.85E-01 | 9.25E-01 |
| PE(P-18:0/18:2) FA | -0.5 | -50 | 49 | 9.84E-01 | 9.98E-01 | -12.3 | -76 | 51 | 7.03E-01 | 9.01E-01 | 24.2 | -52 | 100 | 5.34E-01 | 7.98E-01 |
| PE(P-18:0/20:4) FA | 14.9 | -37 | 67 | 5.76E-01 | 9.98E-01 | -3.6 | -72 | 65 | 9.17E-01 | 9.55E-01 | 23.0 | -61 | 107 | 5.94E-01 | 8.35E-01 |
| PE(P-18:0/22:5) FA | 21.6 | -27 | 70 | 3.85E-01 | 9.98E-01 | 1.0 | -62 | 64 | 9.75E-01 | 9.88E-01 | 14.2 | -58 | 86 | 7.00E-01 | 8.90E-01 |
| PE(P-18:0/22:6) FA | -14.4 | -64 | 35 | 5.68E-01 | 9.98E-01 | -26.4 | -81 | 29 | 3.50E-01 | 7.11E-01 | 18.9 | -54 | 91 | 6.11E-01 | 8.39E-01 |
| PE(P-20:0/20:4) FA | -12.6 | -66 | 41 | 6.48E-01 | 9.98E-01 | -13.2 | -85 | 58 | 7.19E-01 | 9.03E-01 | -34.8 | -117 | 48 | 4.09E-01 | 7.01E-01 |
| PG 34:1 | -64.7 | -128 | -2 | **4.65E-02** | 9.98E-01 | -112.3 | -191 | -33 | **6.40E-03** | 7.21E-02 | -85.5 | -181 | 10 | 8.22E-02 | 3.67E-01 |
| PG 36:1 | -11.4 | -64 | 42 | 6.74E-01 | 9.98E-01 | -75.8 | -149 | -3 | **4.53E-02** | 2.54E-01 | -72.7 | -160 | 15 | 1.07E-01 | 3.95E-01 |
| PG 36:2 | -15.7 | -70 | 39 | 5.73E-01 | 9.98E-01 | -69.7 | -138 | -2 | **4.70E-02** | 2.59E-01 | -42.5 | -119 | 34 | 2.78E-01 | 5.80E-01 |
| LPE 16:0 | -57.9 | -117 | 1 | 5.58E-02 | 9.98E-01 | -49.2 | -120 | 22 | 1.79E-01 | 5.01E-01 | -74.5 | -170 | 21 | 1.28E-01 | 4.24E-01 |
| LPE 18:0 | -19.3 | -64 | 26 | 4.04E-01 | 9.98E-01 | -26.0 | -87 | 35 | 4.03E-01 | 7.52E-01 | -63.0 | -136 | 10 | 9.49E-02 | 3.85E-01 |
| LPE 18:1 | 24.5 | -38 | 87 | 4.47E-01 | 9.98E-01 | 54.9 | -29 | 139 | 2.03E-01 | 5.38E-01 | 91.2 | -14 | 196 | 9.13E-02 | 3.85E-01 |
| LPE 18:2 | -2.6 | -64 | 59 | 9.33E-01 | 9.98E-01 | 30.5 | -46 | 107 | 4.37E-01 | 7.75E-01 | 96.0 | 0 | 192 | 5.16E-02 | 2.78E-01 |
| LPE 20:4 | 10.3 | -43 | 63 | 7.04E-01 | 9.98E-01 | 10.2 | -65 | 86 | 7.91E-01 | 9.27E-01 | -11.9 | -95 | 72 | 7.80E-01 | 9.25E-01 |
| LPE 22:6 | -16.4 | -73 | 40 | 5.71E-01 | 9.98E-01 | -35.9 | -113 | 42 | 3.66E-01 | 7.26E-01 | -51.7 | -144 | 40 | 2.72E-01 | 5.80E-01 |
| PI 32:0 | -11.1 | -70 | 48 | 7.15E-01 | 9.98E-01 | -43.1 | -127 | 40 | 3.14E-01 | 6.61E-01 | 66.2 | -29 | 162 | 1.77E-01 | 4.95E-01 |
| PI 32:1 | 8.0 | -48 | 64 | 7.79E-01 | 9.98E-01 | -103.9 | -187 | -21 | **1.62E-02** | 1.21E-01 | -11.5 | -96 | 73 | 7.91E-01 | 9.25E-01 |
| PI 34:0 | -20.2 | -84 | 44 | 5.40E-01 | 9.98E-01 | -12.1 | -100 | 76 | 7.89E-01 | 9.27E-01 | 88.2 | -16 | 192 | 9.97E-02 | 3.95E-01 |
| PI 34:1 | 18.7 | -44 | 81 | 5.60E-01 | 9.98E-01 | -22.5 | -104 | 59 | 5.88E-01 | 8.73E-01 | 45.5 | -54 | 145 | 3.71E-01 | 6.80E-01 |
| PI 36:1 | 33.0 | -20 | 86 | 2.25E-01 | 9.98E-01 | 48.7 | -23 | 120 | 1.86E-01 | 5.05E-01 | 93.0 | 9 | 178 | **3.33E-02** | 2.34E-01 |
| PI 36:2 | 8.0 | -47 | 63 | 7.76E-01 | 9.98E-01 | 32.2 | -32 | 96 | 3.26E-01 | 6.73E-01 | 69.3 | -18 | 157 | 1.23E-01 | 4.20E-01 |
| PI 36:3 | 8.5 | -38 | 55 | 7.18E-01 | 9.98E-01 | 15.0 | -45 | 75 | 6.26E-01 | 8.81E-01 | 64.4 | -4 | 133 | 6.79E-02 | 3.42E-01 |
| PI 36:4 | -0.2 | -60 | 59 | 9.96E-01 | 9.98E-01 | -58.7 | -130 | 13 | 1.12E-01 | 3.98E-01 | -7.6 | -101 | 86 | 8.73E-01 | 9.59E-01 |
| PI 38:2 | -6.1 | -66 | 54 | 8.43E-01 | 9.98E-01 | 3.2 | -78 | 85 | 9.40E-01 | 9.73E-01 | 60.8 | -34 | 155 | 2.10E-01 | 5.21E-01 |
| PI 38:3 | -26.7 | -78 | 25 | 3.12E-01 | 9.98E-01 | -22.8 | -85 | 39 | 4.73E-01 | 8.02E-01 | -1.4 | -83 | 80 | 9.73E-01 | 9.90E-01 |
| PI 38:4 | -19.6 | -82 | 43 | 5.39E-01 | 9.98E-01 | -11.6 | -98 | 75 | 7.93E-01 | 9.27E-01 | -22.7 | -123 | 78 | 6.60E-01 | 8.65E-01 |
| PI 38:5 | -6.6 | -60 | 47 | 8.09E-01 | 9.98E-01 | -18.5 | -90 | 53 | 6.11E-01 | 8.81E-01 | 39.3 | -47 | 126 | 3.73E-01 | 6.80E-01 |
| PI 38:6 | -2.0 | -48 | 44 | 9.32E-01 | 9.98E-01 | -17.4 | -80 | 45 | 5.89E-01 | 8.73E-01 | 17.0 | -58 | 92 | 6.58E-01 | 8.65E-01 |
| PI 40:4 | 8.3 | -48 | 65 | 7.75E-01 | 9.98E-01 | -30.6 | -107 | 46 | 4.35E-01 | 7.75E-01 | -23.4 | -112 | 65 | 6.05E-01 | 8.35E-01 |
| PI 40:5 | -7.4 | -59 | 44 | 7.76E-01 | 9.98E-01 | -38.5 | -103 | 26 | 2.44E-01 | 5.94E-01 | -28.1 | -104 | 48 | 4.69E-01 | 7.52E-01 |
| PI 40:6 | -14.3 | -61 | 33 | 5.52E-01 | 9.98E-01 | -31.7 | -90 | 26 | 2.87E-01 | 6.45E-01 | -10.9 | -90 | 68 | 7.88E-01 | 9.25E-01 |
| LPI 18:0 | 3.0 | -53 | 59 | 9.16E-01 | 9.98E-01 | 14.0 | -60 | 88 | 7.13E-01 | 9.01E-01 | -34.9 | -120 | 50 | 4.24E-01 | 7.18E-01 |
| LPI 18:1 | 38.3 | -20 | 96 | 1.98E-01 | 9.98E-01 | 72.3 | 1 | 144 | 5.07E-02 | 2.65E-01 | 96.6 | 8 | 185 | **3.53E-02** | 2.35E-01 |
| LPI 18:2 | -5.1 | -55 | 44 | 8.40E-01 | 9.98E-01 | 59.4 | -3 | 122 | 6.71E-02 | 3.13E-01 | 70.0 | -6 | 146 | 7.23E-02 | 3.50E-01 |
| LPI 20:4 | -36.8 | -96 | 23 | 2.29E-01 | 9.98E-01 | 4.8 | -69 | 78 | 8.99E-01 | 9.55E-01 | 12.6 | -74 | 99 | 7.75E-01 | 9.25E-01 |
| PS 36:1 | 7.0 | -38 | 52 | 7.60E-01 | 9.98E-01 | -18.8 | -83 | 45 | 5.66E-01 | 8.64E-01 | -54.1 | -121 | 13 | 1.18E-01 | 4.14E-01 |
| PS 36:2 | 1.7 | -50 | 53 | 9.49E-01 | 9.98E-01 | -21.9 | -92 | 48 | 5.40E-01 | 8.47E-01 | -41.3 | -117 | 35 | 2.88E-01 | 5.85E-01 |
| PS 38:3 | -0.2 | -47 | 46 | 9.93E-01 | 9.98E-01 | -16.4 | -77 | 44 | 5.94E-01 | 8.73E-01 | -42.9 | -114 | 28 | 2.41E-01 | 5.68E-01 |
| PS 38:4 | 0.0 | -46 | 46 | 9.98E-01 | 9.98E-01 | -32.0 | -92 | 28 | 2.98E-01 | 6.52E-01 | -47.1 | -113 | 19 | 1.65E-01 | 4.85E-01 |
| PS 38:5 | -2.8 | -52 | 46 | 9.12E-01 | 9.98E-01 | -16.6 | -77 | 44 | 5.91E-01 | 8.73E-01 | -24.2 | -97 | 48 | 5.13E-01 | 7.81E-01 |
| PS 40:5 | 3.7 | -47 | 54 | 8.85E-01 | 9.98E-01 | -35.8 | -106 | 34 | 3.20E-01 | 6.68E-01 | -54.7 | -130 | 20 | 1.57E-01 | 4.69E-01 |
| PS 40:6 | -15.6 | -68 | 37 | 5.59E-01 | 9.98E-01 | -54.0 | -118 | 10 | 1.02E-01 | 3.95E-01 | -71.9 | -144 | 0 | 5.20E-02 | 2.78E-01 |
| COH | 1.8 | -59 | 63 | 9.54E-01 | 9.98E-01 | 19.1 | -57 | 95 | 6.23E-01 | 8.81E-01 | -15.8 | -105 | 74 | 7.30E-01 | 8.98E-01 |
| CE 14:0 | -1.7 | -60 | 56 | 9.53E-01 | 9.98E-01 | -101.2 | -198 | -4 | **4.32E-02** | 2.50E-01 | -8.5 | -97 | 80 | 8.52E-01 | 9.54E-01 |
| CE 15:0 | -2.9 | -53 | 47 | 9.10E-01 | 9.98E-01 | -12.3 | -76 | 52 | 7.07E-01 | 9.01E-01 | 11.4 | -70 | 93 | 7.83E-01 | 9.25E-01 |
| CE 16:0 | -23.4 | -88 | 41 | 4.77E-01 | 9.98E-01 | -71.9 | -152 | 8 | 8.15E-02 | 3.50E-01 | -83.4 | -183 | 17 | 1.05E-01 | 3.95E-01 |
| CE 16:1 | -4.8 | -64 | 55 | 8.74E-01 | 9.98E-01 | -149.9 | -228 | -72 | **2.77E-04** | **1.09E-02** | -110.2 | -196 | -24 | **1.37E-02** | 1.59E-01 |
| CE 16:2 | -15.5 | -74 | 43 | 6.06E-01 | 9.98E-01 | -112.2 | -193 | -32 | **7.56E-03** | 7.85E-02 | -63.4 | -153 | 26 | 1.68E-01 | 4.87E-01 |
| CE 17:0 | -27.1 | -76 | 22 | 2.78E-01 | 9.98E-01 | -55.1 | -113 | 3 | 6.74E-02 | 3.13E-01 | -47.2 | -118 | 24 | 1.96E-01 | 5.06E-01 |
| CE 17:1 | 4.2 | -49 | 57 | 8.78E-01 | 9.98E-01 | -56.0 | -132 | 20 | 1.52E-01 | 4.45E-01 | -23.7 | -107 | 59 | 5.77E-01 | 8.29E-01 |
| CE 18:0 | 0.8 | -52 | 54 | 9.77E-01 | 9.98E-01 | -37.4 | -106 | 32 | 2.90E-01 | 6.45E-01 | -49.0 | -134 | 36 | 2.59E-01 | 5.71E-01 |
| CE 18:1 | -54.5 | -116 | 7 | 8.73E-02 | 9.98E-01 | -134.1 | -214 | -55 | **1.31E-03** | **2.88E-02** | -116.0 | -207 | -25 | **1.44E-02** | 1.59E-01 |
| CE 18:2 | -66.5 | -123 | -10 | **2.26E-02** | 9.98E-01 | -153.7 | -228 | -79 | **1.06E-04** | **6.07E-03** | -136.0 | -223 | -49 | **2.93E-03** | 9.41E-02 |
| CE 18:3 | -26.1 | -91 | 38 | 4.30E-01 | 9.98E-01 | -142.7 | -221 | -64 | **5.85E-04** | **2.07E-02** | -114.4 | -203 | -26 | **1.26E-02** | 1.59E-01 |
| CE 20:1 | -36.2 | -82 | 10 | 1.26E-01 | 9.98E-01 | -24.4 | -81 | 33 | 4.02E-01 | 7.52E-01 | -29.0 | -94 | 36 | 3.85E-01 | 6.97E-01 |
| CE 20:2 | -25.0 | -88 | 38 | 4.37E-01 | 9.98E-01 | -82.5 | -162 | -3 | **4.44E-02** | 2.53E-01 | -78.6 | -178 | 21 | 1.26E-01 | 4.24E-01 |
| CE 20:3 | -0.3 | -64 | 63 | 9.93E-01 | 9.98E-01 | -101.4 | -180 | -23 | **1.33E-02** | 1.09E-01 | -90.2 | -182 | 2 | 5.69E-02 | 2.91E-01 |
| CE 20:4 | -18.6 | -74 | 37 | 5.11E-01 | 9.98E-01 | -77.4 | -144 | -10 | **2.55E-02** | 1.70E-01 | -96.5 | -180 | -13 | **2.63E-02** | 2.18E-01 |
| CE 20:5 | -8.7 | -53 | 35 | 6.98E-01 | 9.98E-01 | -46.1 | -105 | 13 | 1.29E-01 | 4.25E-01 | -39.1 | -109 | 30 | 2.73E-01 | 5.80E-01 |
| CE 22:0 | -40.5 | -74 | -7 | **2.11E-02** | 9.98E-01 | -42.9 | -89 | 3 | 6.89E-02 | 3.13E-01 | -89.7 | -145 | -35 | **1.93E-03** | 7.80E-02 |
| CE 22:1 | -18.3 | -58 | 21 | 3.66E-01 | 9.98E-01 | -17.1 | -65 | 31 | 4.84E-01 | 8.09E-01 | -26.9 | -83 | 30 | 3.53E-01 | 6.62E-01 |
| CE 22:4 | -11.3 | -52 | 29 | 5.85E-01 | 9.98E-01 | -45.6 | -99 | 8 | 9.91E-02 | 3.95E-01 | -55.6 | -118 | 6 | 8.14E-02 | 3.67E-01 |
| CE 22:5 | -24.3 | -77 | 29 | 3.70E-01 | 9.98E-01 | -93.6 | -157 | -30 | **4.91E-03** | 6.26E-02 | -98.3 | -179 | -18 | **1.87E-02** | 1.81E-01 |
| CE 22:6 | -43.8 | -93 | 6 | 8.55E-02 | 9.98E-01 | -96.1 | -161 | -31 | **4.64E-03** | 6.26E-02 | -112.7 | -191 | -35 | **5.61E-03** | 1.19E-01 |
| CE 24:0 | -31.8 | -74 | 11 | 1.44E-01 | 9.98E-01 | -59.3 | -113 | -6 | **3.27E-02** | 1.96E-01 | -102.9 | -167 | -39 | **2.21E-03** | 7.80E-02 |
| CE 24:1 | -18.7 | -80 | 42 | 5.50E-01 | 9.98E-01 | -38.1 | -112 | 35 | 3.13E-01 | 6.61E-01 | -65.7 | -155 | 24 | 1.54E-01 | 4.69E-01 |
| CE 24:4 | 6.7 | -33 | 46 | 7.41E-01 | 9.98E-01 | -45.8 | -105 | 13 | 1.30E-01 | 4.25E-01 | -27.8 | -90 | 35 | 3.87E-01 | 6.97E-01 |
| CE 24:5 | 11.0 | -41 | 63 | 6.81E-01 | 9.98E-01 | -50.7 | -117 | 15 | 1.36E-01 | 4.31E-01 | -21.8 | -103 | 60 | 6.01E-01 | 8.35E-01 |
| CE 24:6 | 22.2 | -32 | 76 | 4.24E-01 | 9.98E-01 | -58.3 | -131 | 14 | 1.18E-01 | 4.04E-01 | -56.4 | -141 | 28 | 1.95E-01 | 5.06E-01 |
| DG 14:0/16:0 | -22.5 | -75 | 30 | 4.01E-01 | 9.98E-01 | -117.6 | -196 | -39 | **4.07E-03** | 6.26E-02 | -26.2 | -106 | 54 | 5.23E-01 | 7.90E-01 |
| DG 14:0/16:1 | -35.1 | -84 | 14 | 1.60E-01 | 9.98E-01 | -171.1 | -242 | -100 | **6.96E-06** | **2.46E-03** | -72.7 | -156 | 11 | 9.00E-02 | 3.85E-01 |
| DG 14:0/18:1 | -26.4 | -83 | 31 | 3.66E-01 | 9.98E-01 | -111.7 | -183 | -40 | **2.86E-03** | 5.53E-02 | -39.4 | -117 | 39 | 3.24E-01 | 6.35E-01 |
| DG 14:0/18:2 | -19.9 | -67 | 28 | 4.13E-01 | 9.98E-01 | -75.2 | -145 | -5 | **3.76E-02** | 2.21E-01 | 3.1 | -70 | 76 | 9.34E-01 | 9.81E-01 |
| DG 16:0/16:0 | -26.1 | -76 | 24 | 3.10E-01 | 9.98E-01 | -63.9 | -131 | 3 | 6.38E-02 | 3.10E-01 | -66.4 | -146 | 13 | 1.07E-01 | 3.95E-01 |
| DG 16:0/16:1 | -46.0 | -103 | 11 | 1.15E-01 | 9.98E-01 | -153.6 | -222 | -85 | **2.92E-05** | **3.44E-03** | -138.2 | -221 | -55 | **1.47E-03** | 7.41E-02 |
| DG 16:0/18:1 | -49.6 | -105 | 6 | 8.16E-02 | 9.98E-01 | -127.1 | -198 | -56 | **6.80E-04** | **2.18E-02** | -119.2 | -203 | -36 | **6.09E-03** | 1.19E-01 |
| DG 16:0/18:1 | -51.5 | -112 | 9 | 9.85E-02 | 9.98E-01 | -136.9 | -215 | -59 | **8.39E-04** | **2.40E-02** | -118.8 | -211 | -27 | **1.32E-02** | 1.59E-01 |
| DG 16:0/18:2 | -49.2 | -102 | 4 | 7.27E-02 | 9.98E-01 | -87.8 | -159 | -17 | **1.76E-02** | 1.26E-01 | -86.0 | -169 | -3 | **4.61E-02** | 2.70E-01 |
| DG 16:0/20:3 | -5.7 | -70 | 59 | 8.63E-01 | 9.98E-01 | -63.9 | -140 | 13 | 1.05E-01 | 3.96E-01 | -57.7 | -157 | 42 | 2.58E-01 | 5.71E-01 |
| DG 16:0/20:4 | 28.4 | -16 | 73 | 2.10E-01 | 9.98E-01 | -10.6 | -70 | 49 | 7.27E-01 | 9.10E-01 | 14.8 | -53 | 83 | 6.71E-01 | 8.65E-01 |
| DG 16:0/22:5 | -43.2 | -93 | 7 | 9.25E-02 | 9.98E-01 | -90.8 | -160 | -22 | **1.16E-02** | 1.03E-01 | -49.3 | -133 | 35 | 2.52E-01 | 5.69E-01 |
| DG 16:0/22:6 | -13.2 | -55 | 29 | 5.38E-01 | 9.98E-01 | -49.8 | -103 | 4 | 7.13E-02 | 3.19E-01 | -24.4 | -90 | 42 | 4.71E-01 | 7.52E-01 |
| DG 16:1/16:1 | -41.4 | -99 | 16 | 1.62E-01 | 9.98E-01 | -175.9 | -254 | -97 | **2.80E-05** | **3.44E-03** | -170.4 | -258 | -83 | **2.41E-04** | **3.20E-02** |
| DG 16:1/18:1 | -54.3 | -116 | 7 | 8.61E-02 | 9.98E-01 | -156.4 | -230 | -82 | **7.19E-05** | **6.07E-03** | -162.3 | -249 | -75 | **4.22E-04** | **3.20E-02** |
| DG 18:0/18:1 | -26.9 | -94 | 40 | 4.31E-01 | 9.98E-01 | -107.1 | -186 | -28 | **9.37E-03** | 9.45E-02 | -119.1 | -213 | -26 | **1.41E-02** | 1.59E-01 |
| DG 18:0/18:2 | -41.8 | -105 | 22 | 2.01E-01 | 9.98E-01 | -92.3 | -175 | -10 | **3.05E-02** | 1.89E-01 | -77.3 | -176 | 21 | 1.28E-01 | 4.24E-01 |
| DG 18:0/20:4 | 29.7 | -29 | 88 | 3.23E-01 | 9.98E-01 | -43.0 | -121 | 35 | 2.84E-01 | 6.44E-01 | -95.2 | -188 | -2 | **4.76E-02** | 2.71E-01 |
| DG 18:1/18:1 | -51.0 | -110 | 8 | 9.28E-02 | 9.98E-01 | -128.5 | -202 | -55 | **8.83E-04** | **2.40E-02** | -160.8 | -248 | -74 | **4.53E-04** | **3.20E-02** |
| DG 18:1/18:2 | -48.8 | -98 | 1 | 5.57E-02 | 9.98E-01 | -90.0 | -154 | -26 | **6.54E-03** | 7.21E-02 | -107.1 | -181 | -33 | **5.61E-03** | 1.19E-01 |
| DG 18:1/18:3 | -33.1 | -86 | 19 | 2.20E-01 | 9.98E-01 | -108.6 | -182 | -36 | **4.43E-03** | 6.26E-02 | -122.7 | -210 | -35 | **7.04E-03** | 1.21E-01 |
| DG 18:1/20:3 | 26.4 | -29 | 82 | 3.52E-01 | 9.98E-01 | -30.1 | -101 | 41 | 4.08E-01 | 7.55E-01 | -54.1 | -135 | 27 | 1.95E-01 | 5.06E-01 |
| DG 18:1/20:4 | 36.4 | -10 | 83 | 1.29E-01 | 9.98E-01 | -11.3 | -70 | 47 | 7.05E-01 | 9.01E-01 | -20.9 | -99 | 57 | 5.99E-01 | 8.35E-01 |
| DG 18:2/18:2 | -30.7 | -70 | 9 | 1.32E-01 | 9.98E-01 | -11.8 | -63 | 39 | 6.51E-01 | 8.91E-01 | -16.5 | -78 | 44 | 5.96E-01 | 8.35E-01 |
| DG 18:2/20:3 | 47.0 | -10 | 104 | 1.08E-01 | 9.98E-01 | 18.1 | -53 | 89 | 6.18E-01 | 8.81E-01 | 0.6 | -80 | 81 | 9.88E-01 | 9.94E-01 |
| DG 18:2/20:4 | 23.9 | -20 | 68 | 2.90E-01 | 9.98E-01 | 20.9 | -39 | 80 | 4.93E-01 | 8.14E-01 | 27.8 | -44 | 99 | 4.47E-01 | 7.32E-01 |
| TG 14:0/16:0/18:1 | -24.5 | -77 | 28 | 3.60E-01 | 9.98E-01 | -99.2 | -176 | -23 | **1.23E-02** | 1.03E-01 | -18.0 | -103 | 67 | 6.77E-01 | 8.69E-01 |
| TG 14:0/16:0/18:2 | -20.1 | -73 | 33 | 4.60E-01 | 9.98E-01 | -88.8 | -157 | -21 | **1.21E-02** | 1.03E-01 | -36.9 | -124 | 50 | 4.06E-01 | 7.01E-01 |
| TG 14:0/16:1/18:1 | -31.3 | -87 | 25 | 2.76E-01 | 9.98E-01 | -111.5 | -190 | -33 | **6.30E-03** | 7.21E-02 | -50.0 | -135 | 35 | 2.53E-01 | 5.69E-01 |
| TG 14:0/16:1/18:2 | -18.0 | -62 | 26 | 4.27E-01 | 9.98E-01 | -85.6 | -151 | -20 | **1.17E-02** | 1.03E-01 | -30.0 | -101 | 41 | 4.08E-01 | 7.01E-01 |
| TG 14:0/17:0/18:1 | -20.1 | -78 | 38 | 5.00E-01 | 9.98E-01 | -60.2 | -143 | 22 | 1.57E-01 | 4.50E-01 | -36.6 | -127 | 54 | 4.29E-01 | 7.18E-01 |
| TG 14:0/18:0/18:1 | 6.6 | -56 | 70 | 8.37E-01 | 9.98E-01 | -40.0 | -126 | 46 | 3.64E-01 | 7.26E-01 | 6.5 | -88 | 101 | 8.93E-01 | 9.61E-01 |
| TG 14:0/18:2/18:2 | -22.8 | -74 | 28 | 3.84E-01 | 9.98E-01 | -64.5 | -132 | 3 | 6.42E-02 | 3.10E-01 | -30.1 | -109 | 49 | 4.59E-01 | 7.44E-01 |
| TG 14:1/16:0/18:1 | -18.8 | -71 | 34 | 4.86E-01 | 9.98E-01 | -81.9 | -147 | -17 | **1.56E-02** | 1.20E-01 | -41.7 | -114 | 31 | 2.64E-01 | 5.77E-01 |
| TG 14:1/16:1/18:0 | -38.5 | -92 | 15 | 1.65E-01 | 9.98E-01 | -144.9 | -215 | -74 | **1.10E-04** | **6.07E-03** | -110.9 | -197 | -25 | **1.31E-02** | 1.59E-01 |
| TG 14:1/18:0/18:2 | -22.7 | -76 | 31 | 4.10E-01 | 9.98E-01 | -78.4 | -144 | -13 | **2.08E-02** | 1.47E-01 | -77.2 | -165 | 11 | 8.80E-02 | 3.83E-01 |
| TG 14:1/18:1/18:1 | -40.6 | -99 | 18 | 1.75E-01 | 9.98E-01 | -111.1 | -183 | -40 | **2.97E-03** | 5.53E-02 | -113.0 | -199 | -27 | **1.19E-02** | 1.59E-01 |
| TG 15:0/16:0/18:1 | -17.7 | -78 | 42 | 5.65E-01 | 9.98E-01 | -67.5 | -148 | 13 | 1.04E-01 | 3.96E-01 | -60.5 | -153 | 32 | 2.05E-01 | 5.16E-01 |
| TG 15:0/18:1/18:1 | -55.3 | -122 | 12 | 1.09E-01 | 9.98E-01 | -132.0 | -220 | -44 | **3.94E-03** | 6.26E-02 | -134.2 | -233 | -35 | **9.25E-03** | 1.48E-01 |
| TG 16:0/16:0/16:0 | -13.1 | -70 | 44 | 6.54E-01 | 9.98E-01 | -69.8 | -141 | 2 | 5.91E-02 | 3.02E-01 | -11.1 | -106 | 84 | 8.20E-01 | 9.46E-01 |
| TG 16:0/16:0/18:0 | -9.7 | -66 | 47 | 7.37E-01 | 9.98E-01 | -45.3 | -117 | 26 | 2.18E-01 | 5.54E-01 | -34.7 | -121 | 52 | 4.33E-01 | 7.18E-01 |
| TG 16:0/16:0/18:1 | -40.9 | -93 | 11 | 1.27E-01 | 9.98E-01 | -100.4 | -168 | -33 | **4.60E-03** | 6.26E-02 | -75.6 | -157 | 6 | 7.10E-02 | 3.50E-01 |
| TG 16:0/16:0/18:2 | -52.9 | -106 | 0 | 5.26E-02 | 9.98E-01 | -101.3 | -178 | -24 | **1.12E-02** | 1.03E-01 | -69.0 | -153 | 15 | 1.12E-01 | 4.00E-01 |
| TG 16:0/16:1/17:0 | -18.7 | -76 | 38 | 5.22E-01 | 9.98E-01 | -83.0 | -165 | -1 | 5.10E-02 | 2.65E-01 | -46.7 | -142 | 49 | 3.39E-01 | 6.45E-01 |
| TG 16:0/16:1/18:1 | -46.1 | -118 | 26 | 2.10E-01 | 9.98E-01 | -156.7 | -247 | -66 | **9.88E-04** | **2.49E-02** | -144.7 | -247 | -42 | **6.86E-03** | 1.21E-01 |
| TG 16:0/17:0/18:0 | -1.3 | -61 | 58 | 9.65E-01 | 9.98E-01 | -60.0 | -140 | 20 | 1.46E-01 | 4.45E-01 | -60.2 | -151 | 30 | 1.96E-01 | 5.06E-01 |
| TG 16:0/17:0/18:1 | -4.5 | -55 | 46 | 8.61E-01 | 9.98E-01 | -53.7 | -125 | 17 | 1.42E-01 | 4.43E-01 | -51.9 | -138 | 34 | 2.42E-01 | 5.68E-01 |
| TG 16:0/17:0/18:2 | -19.3 | -80 | 41 | 5.35E-01 | 9.98E-01 | -99.5 | -176 | -23 | **1.19E-02** | 1.03E-01 | -106.7 | -194 | -20 | **1.83E-02** | 1.81E-01 |
| TG 16:0/18:0/18:1 | -12.8 | -58 | 32 | 5.76E-01 | 9.98E-01 | -60.1 | -112 | -8 | **2.49E-02** | 1.69E-01 | -47.9 | -110 | 14 | 1.35E-01 | 4.31E-01 |
| TG 16:0/18:1/18:1 | -33.2 | -94 | 27 | 2.86E-01 | 9.98E-01 | -126.8 | -212 | -42 | **4.30E-03** | 6.26E-02 | -144.4 | -239 | -50 | **3.57E-03** | 1.05E-01 |
| TG 16:0/18:1/18:2 | -41.0 | -103 | 21 | 2.00E-01 | 9.98E-01 | -102.9 | -183 | -23 | **1.36E-02** | 1.09E-01 | -139.6 | -227 | -53 | **2.16E-03** | 7.80E-02 |
| TG 16:0/18:2/18:2 | -44.0 | -95 | 7 | 9.50E-02 | 9.98E-01 | -57.3 | -119 | 4 | 6.93E-02 | 3.13E-01 | -85.3 | -164 | -6 | **3.66E-02** | 2.35E-01 |
| TG 16:1/16:1/16:1 | -34.8 | -84 | 14 | 1.65E-01 | 9.98E-01 | -140.2 | -212 | -68 | **2.33E-04** | **1.03E-02** | -112.0 | -196 | -28 | **1.02E-02** | 1.57E-01 |
| TG 16:1/16:1/18:0 | -9.6 | -67 | 48 | 7.44E-01 | 9.98E-01 | -68.4 | -146 | 9 | 8.72E-02 | 3.63E-01 | -26.8 | -116 | 63 | 5.58E-01 | 8.18E-01 |
| TG 16:1/16:1/18:1 | -44.3 | -102 | 14 | 1.38E-01 | 9.98E-01 | -163.2 | -243 | -83 | **1.20E-04** | **6.07E-03** | -177.8 | -268 | -88 | **1.98E-04** | **3.20E-02** |
| TG 16:1/17:0/18:1 | -32.3 | -97 | 33 | 3.32E-01 | 9.98E-01 | -106.7 | -193 | -21 | **1.66E-02** | 1.22E-01 | -109.1 | -206 | -13 | **2.91E-02** | 2.18E-01 |
| TG 16:1/18:1/18:1 | -37.8 | -90 | 14 | 1.57E-01 | 9.98E-01 | -110.8 | -181 | -40 | **2.61E-03** | 5.41E-02 | -149.2 | -230 | -69 | **4.43E-04** | **3.20E-02** |
| TG 16:1/18:1/18:2 | -41.3 | -91 | 9 | 1.07E-01 | 9.98E-01 | -106.1 | -169 | -44 | **1.24E-03** | **2.88E-02** | -126.5 | -199 | -54 | **9.19E-04** | 5.41E-02 |
| TG 17:0/18:1/18:1 | -10.5 | -69 | 48 | 7.27E-01 | 9.98E-01 | -72.9 | -159 | 14 | 1.02E-01 | 3.95E-01 | -111.2 | -208 | -14 | **2.67E-02** | 2.18E-01 |
| TG 18:0/18:0/18:0 | -4.9 | -64 | 54 | 8.70E-01 | 9.98E-01 | -35.6 | -113 | 42 | 3.70E-01 | 7.29E-01 | -39.3 | -120 | 41 | 3.42E-01 | 6.45E-01 |
| TG 18:0/18:0/18:1 | -0.1 | -51 | 51 | 9.95E-01 | 9.98E-01 | -47.7 | -115 | 20 | 1.68E-01 | 4.74E-01 | -57.4 | -136 | 22 | 1.57E-01 | 4.69E-01 |
| TG 18:0/18:1/18:1 | -21.1 | -70 | 27 | 3.96E-01 | 9.98E-01 | -73.9 | -137 | -11 | **2.41E-02** | 1.67E-01 | -91.5 | -165 | -18 | **1.63E-02** | 1.75E-01 |
| TG 18:0/18:2/18:2 | -18.9 | -65 | 27 | 4.22E-01 | 9.98E-01 | -32.5 | -94 | 29 | 3.00E-01 | 6.52E-01 | -40.3 | -111 | 30 | 2.65E-01 | 5.77E-01 |
| TG 18:1/18:1/18:1 | -22.8 | -79 | 33 | 4.29E-01 | 9.98E-01 | -64.1 | -150 | 21 | 1.45E-01 | 4.45E-01 | -101.7 | -202 | -2 | **4.91E-02** | 2.75E-01 |
| TG 18:1/18:1/18:2 | -10.4 | -53 | 33 | 6.37E-01 | 9.98E-01 | -47.1 | -102 | 8 | 9.58E-02 | 3.93E-01 | -74.2 | -140 | -9 | **2.90E-02** | 2.18E-01 |
| TG 18:1/18:1/20:4 | 21.2 | -31 | 73 | 4.25E-01 | 9.98E-01 | -20.5 | -87 | 46 | 5.44E-01 | 8.50E-01 | -63.8 | -146 | 18 | 1.31E-01 | 4.24E-01 |
| TG 18:1/18:1/22:6 | 0.8 | -30 | 31 | 9.60E-01 | 9.98E-01 | -25.3 | -63 | 13 | 1.96E-01 | 5.23E-01 | -24.6 | -75 | 25 | 3.36E-01 | 6.45E-01 |
| TG 18:1/18:2/18:2 | -29.3 | -75 | 16 | 2.07E-01 | 9.98E-01 | -50.8 | -109 | 7 | 8.74E-02 | 3.63E-01 | -46.3 | -112 | 20 | 1.72E-01 | 4.91E-01 |
| TG 18:2/18:2/18:2 | -17.2 | -41 | 7 | 1.64E-01 | 9.98E-01 | -4.9 | -38 | 28 | 7.75E-01 | 9.27E-01 | -0.6 | -40 | 38 | 9.77E-01 | 9.91E-01 |
| TG 18:2/18:2/20:4 | 10.2 | -37 | 57 | 6.72E-01 | 9.98E-01 | 21.9 | -34 | 78 | 4.48E-01 | 7.80E-01 | 15.5 | -56 | 87 | 6.71E-01 | 8.65E-01 |
| CE 18:2 +2O #A | -14.7 | -49 | 19 | 3.96E-01 | 9.98E-01 | -24.0 | -67 | 19 | 2.78E-01 | 6.38E-01 | -33.9 | -93 | 26 | 2.66E-01 | 5.77E-01 |
| CE 18:2 +2O #B | -37.0 | -104 | 30 | 2.82E-01 | 9.98E-01 | -128.7 | -215 | -43 | **4.16E-03** | 6.26E-02 | -147.4 | -248 | -47 | **4.83E-03** | 1.19E-01 |
| CE 18:2 +O NH4 | -16.3 | -64 | 31 | 5.02E-01 | 9.98E-01 | -55.3 | -122 | 11 | 1.07E-01 | 3.96E-01 | -59.8 | -133 | 13 | 1.13E-01 | 4.00E-01 |
| PC 34:2 + 2O | 28.0 | -36 | 92 | 3.94E-01 | 9.98E-01 | 71.8 | -14 | 158 | 1.06E-01 | 3.96E-01 | 111.1 | 13 | 209 | **2.85E-02** | 2.18E-01 |
| PC 34:2 + O/PC(O-34:3) +2O/PC(P-34:2) +2O #A | 45.0 | -19 | 109 | 1.72E-01 | 9.98E-01 | 64.7 | -22 | 151 | 1.47E-01 | 4.45E-01 | 24.6 | -77 | 126 | 6.38E-01 | 8.61E-01 |
| PC 34:2 + O/PC(O-34:3) +2O/PC(P-34:2) +2O #B | 66.6 | 6 | 127 | **3.30E-02** | 9.98E-01 | 113.3 | 36 | 191 | **4.97E-03** | 6.26E-02 | 129.8 | 41 | 218 | **4.98E-03** | 1.19E-01 |
| PC 34:2 + O/PC(O-34:3) +2O/PC(P-34:2) +2O #C | 47.7 | -5 | 100 | 7.85E-02 | 9.98E-01 | 89.4 | 22 | 157 | **1.11E-02** | 1.03E-01 | 114.2 | 35 | 194 | **5.94E-03** | 1.19E-01 |

*Associated with an IQR increase in predictor (1 month lipid) concentration
