# Supplementary material for: Plasma lipid species at type 1 diabetes onset predict residual beta-cell function after 6 months
Source: Metabolomics. 2018 Dec 4;14(12):158. doi: 10.1007/s11306-018-1456-3 (PMC6280838; doi:10.1007/s11306-018-1456-3)
Supplement: Supplementary file 3 — Supplementary material 3 (DOCX 69 KB) [file 11306_2018_1456_MOESM3_ESM.docx]

Supplementary figure 1: Principal component analysis (PCA) plot of the QC samples.


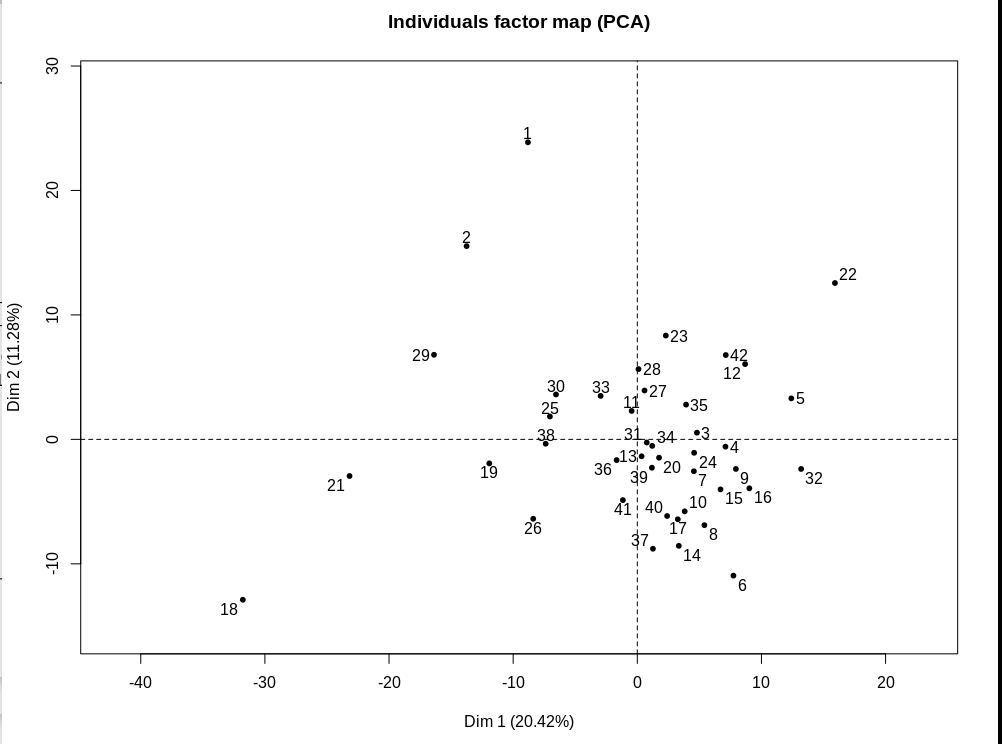


Supplementary figure 1: Visualization of the lipidomic data of the QC samples 1-42 distributed through out the analysis sequence of the samples, using PCA plot.
